# Transmitter and ion channel profiles of neurons in the primate abducens and trochlear nuclei

**DOI:** 10.1007/s00429-021-02315-7

**Published:** 2021-06-28

**Authors:** Ümit Suat Mayadali, Jérome Fleuriet, Michael Mustari, Hans Straka, Anja Kerstin Ellen Horn

**Affiliations:** 1 Institute of Anatomy and Cell Biology, Dept. I, Ludwig-Maximilians-University Munich, Pettenkoferstrasse 11, 80336 Munich, Germany; 2 Graduate School of Systemic Neurosciences, Ludwig-Maximilians-University Munich, Planegg-Martinsried, Germany; 3 Washington National Primate Research Center, Department of Ophthalmology, University of Washington Seattle, Seattle, WA USA; 4 Intensive Care Unit, Raymond Poincaré Hospital, Assistance Publique-Hôpitaux de Paris, Garches, France; 5 Department of Biology II, Ludwig-Maximilians-University Munich, Planegg-Martinsried, Germany

**Keywords:** Voltage-gated potassium channels, Low-voltage activated calcium channels, Glutamate, GABA, Glycine, Extraocular motoneurons, Internuclear neurons

## Abstract

Extraocular motoneurons initiate dynamically different eye movements, including saccades, smooth pursuit and vestibulo-ocular reflexes. These motoneurons subdivide into two main types based on the structure of the neuro-muscular interface: motoneurons of singly-innervated (SIF), and motoneurons of multiply-innervated muscle fibers (MIF). SIF motoneurons are thought to provoke strong and brief/fast muscle contractions, whereas MIF motoneurons initiate prolonged, slow contractions. While relevant for adequate functionality, transmitter and ion channel profiles associated with the morpho-physiological differences between these motoneuron types, have not been elucidated so far. This prompted us to investigate the expression of voltage-gated potassium, sodium and calcium ion channels (*Kv1.1*, *Kv3.1b*, *Nav1.6*, *Cav3.1*–*3.3*, *KCC2*), the transmitter profiles of their presynaptic terminals (*vGlut1* and *2*, *GlyT2* and *GAD*) and transmitter receptors (*GluR2/3*, *NMDAR1*, *GlyR1α*) using immunohistochemical analyses of abducens and trochlear motoneurons and of abducens internuclear neurons (INTs) in macaque monkeys. The main findings were: (1) MIF and SIF motoneurons express unique voltage-gated ion channel profiles, respectively, likely accounting for differences in intrinsic membrane properties. (2) Presynaptic glutamatergic synapses utilize vGlut2, but not vGlut1. (3) Trochlear motoneurons receive GABAergic inputs, abducens neurons receive both GABAergic and glycinergic inputs. (4) Synaptic densities differ between MIF and SIF motoneurons, with MIF motoneurons receiving fewer terminals. (5) Glutamatergic receptor subtypes differ between MIF and SIF motoneurons. While NMDAR1 is intensely expressed in INTs, MIF motoneurons lack this receptor subtype entirely. The obtained cell-type-specific transmitter and conductance profiles illuminate the structural substrates responsible for differential contributions of neurons in the abducens and trochlear nuclei to eye movements.

## Introduction

### Extraocular muscles and innervation by motoneurons in the abducens and trochlear nuclei

Extraocular muscles are responsible for diverse types of eye movements including saccades, smooth pursuit, vestibulo-ocular and optokinetic reflexes, and for fixation (Leigh and Zee 2015). The variation of speed and persistence of these eye movements derive from the contraction of distinct muscle fiber types and their endowment with fast or slow myosin heavy chain isoforms, the number of mitochondria in the sarcoplasm, number and distribution of nerve endings along muscle fibers and the activity of associated motoneurons (Hoh 2020; Horn and Straka 2021). Extraocular muscle fibers can be grouped into one of two main categories according to their innervation patterns: Fast-contracting, fatigable twitch type muscle fibers are innervated by a single *en plaque* ending near the middle of the muscle belly (SIF), whereas slow-contracting, non-fatigable non-twitch type muscle fibers are synaptically contacted along the entire length of the fiber by multiple *en grappe* nerve endings (MIF). In addition, MIFs are also associated with another type of nerve terminal, the palisade endings, which are located at the proximal and distal myotendinous junctions. Whether these unique eye muscle specializations have a sensory or motor function is still being debated (Lienbacher and Horn 2012; Zimmermann et al. 2013).

Eye movements result from coordinated contractions of largely synergistic extraocular muscles through task-specific cooperation by MIFs and SIFs. For horizontal eye movements, lateral rectus muscles are activated by motoneurons in the ipsilateral abducens nucleus (nVI), located in the hindbrain pontine tegmentum; and for vertical and torsional eye movements, by motoneurons in the oculomotor (nIII) and trochlear (nIV) nuclei located in the mesencephalon and rostral hindbrain, respectively (Horn and Straka 2021). The abducens nucleus as an entity consists of four distinct neuronal subtypes: cholinergic motoneurons targeting (1) singly- (SIF) and (2) multiply-innervated (MIF) lateral rectus muscle fibers, (3) glutamatergic internuclear neurons (INT) and (3) paramedian tract neurons (PMT) (Horn et al. 2018; Nguyen and Spencer 1999). While MIF and SIF motoneurons collectively elicit eye muscle contraction, INTs provide the concomitant activation of synergistic medial rectus motoneurons in the contralateral nIII for the generation of conjugate eye movements in the horizontal plane (Büttner-Ennever and Akert 1981). Lastly, PMT neurons presumably send an efference copy of premotor commands to the cerebellar floccular region (Büttner-Ennever 1992; Horn et al. 2018).

The trochlear nucleus, which innervates the contralateral superior oblique muscle, forms one of the final motor elements of the vertical/oblique eye movement circuitry and mainly contains MIF and SIF motoneurons, with only a few internuclear neurons (Ugolini et al. 2006). The two distinct MIF and SIF motoneuronal populations in the abducens and trochlear nuclei were initially demonstrated by tract-tracing from distinct muscle target sites (Büttner-Ennever et al. 2001). In both nuclei, SIF and MIF motoneurons can be retrogradely labeled by tracer injections into the belly of the lateral rectus or superior oblique muscle (Büttner-Ennever et al. 2001; Ugolini et al. 2006). On the other hand, MIF motoneurons can be labeled selectively by tracer injections into the myotendinous junctions of both muscles, which exclusively contain *en grappe* endings (Büttner-Ennever et al. 2001; Ugolini et al. 2006). MIF motoneurons are small or medium-sized neurons clustered in a dorsal cap of the trochlear nucleus. In the abducens nucleus, MIF motoneurons, which form up to 20% of the entire motoneuronal population, are more distributed and accumulate at the medial, dorsal and ventral borders (Eberhorn et al. 2005; Horn et al. 2018; Hernández et al. 2019).

### Histochemical and functional segregation of SIF and MIF motoneurons

MIF and SIF motoneurons in the abducens nucleus form distinct functional subgroups based on differential origins of premotor inputs (Ugolini et al. 2006). Rabies virus injection into the belly of the lateral rectus muscle results in retrograde transneuronal labeling of all premotor cell groups, such as the nucleus prepositus hypoglossi, premotor burst neurons in the paramedian pontine reticular formation (PPRF) and dorsal paragigantocellular nucleus; injection into the myotendinous junction results in transneuronal labeling of premotor neurons involved in gaze-holding and smooth pursuit, but fails to outline other premotor neurons, such as saccade-related burst neurons in the PPRF (Ugolini et al. 2006). This suggests that SIF motoneurons are more involved in targeted eye movements, whereas MIF motoneurons are more suitable to stabilize the eyes around the primary position during fixation of a target (Büttner-Ennever et al. 2001; Dean 1996).

Combined tract-tracing and histochemical studies in monkey have outlined major histochemical differences between MIF and SIF motoneurons, which also served to identify homologous neuronal groups in humans (Horn et al. 2008, 2018). Cholinergic SIF motoneurons of all extraocular motor nuclei are ensheathed by a condensed extracellular matrix, called perineuronal nets (PN) and express the calcium-binding protein parvalbumin (PAV), while MIF motoneurons lack both features (Büttner-Ennever 2006; Eberhorn et al. 2005, 2006; Horn et al. 2008). PNs, together with the expression of PAV, are markers for fast-spiking neurons with high metabolic demands (Härtig et al. 1999; Kodama et al. 2020). In this regard, SIF motoneurons and abducens INTs share similar histochemical profiles (Horn et al. 2018). In addition, an electron microscopic study of medial rectus motoneurons demonstrated a differential density of various types of synaptic contacts on MIF versus SIF motoneurons (Erichsen et al. 2014). The rather tonic firing properties of MIF motoneurons, as demonstrated in frogs, support the idea that MIF motoneurons are particularly suitable for slow changes and tonic maintenance of eye position (Dieringer and Precht 1986; Eberhorn et al. 2005).

At variance with a task-separation of SIF and MIF motoneurons, a number of studies in different species have challenged such a functional segregation. For instance, vestibulo-ocular responses of abducens motoneurons in larval *Xenopus laevis* demonstrated that motoneurons are not segregated into two clearly distinct groups in terms of firing characteristics; instead, these neurons rather form a continuum with respect to firing rate and activation threshold (Dietrich et al. 2017). Potentially, these motoneuron populations may also form a gradient with respect to specific glutamatergic receptor subtypes (i.e., NMDA or AMPA) mediating vestibular excitatory inputs (Dietrich et al. 2017). In addition, four different motoneuronal types were identified in the rat oculomotor nucleus with respect to discharge characteristics in vitro, where, however, none of the subgroups corresponds to anatomically identified MIF motoneurons (Nieto-Gonzales et al. 2007). A distinct, task-specific separation has also been challenged based on recordings in alert cats, where both SIF and MIF motoneurons were recruited regardless of the eye movement type. In addition, both types contribute with a burst/tonic discharge pattern to all eye movement behaviors (Hernández et al. 2019). However, MIF motoneurons exhibit lower discharge thresholds, lower eye movement sensitivities and overall reduced firing rate levels (Hernández et al. 2019). Collectively, these results suggest that the duality of MIF and SIF motoneurons based on the structure of the neuro-muscular interface is likely too simplistic, and thus requires further fine-tuning according to presynaptic transmitter profile, postsynaptic receptor identity and ion channel composition.

### Aim of the study

The apparent discrepancy between electrophysiological and anatomical/histochemical findings in regard to MIF and SIF motoneurons prompted us to investigate potential histochemical differences between the two types by analyzing molecular characteristics known to define intrinsic membrane and/or synaptic properties. Therefore, this study aims at investigating differences in excitatory and inhibitory synaptic inputs, transmitter receptors and voltage-gated ion channels using the previously established histochemical identification scheme of classifying neurons in the abducens and trochlear nuclei.

## Materials and methods

### Brain tissue

Brainstem sections of one new macaque (Case M1) and five previously described macaque monkeys (Cases M2–6) were used in this study (Ahlfeld et al. 2011; Lienbacher et al. 2011; Table 1). Frozen sections from three cases (M1–3) and paraffin sections from three other cases (M4–6) were used. All animals had been similarly fixed by transcardial perfusion with 4% paraformaldehyde (PFA) in 0.1 M phosphate buffer (PB; pH 7.4).

**Table 1:**
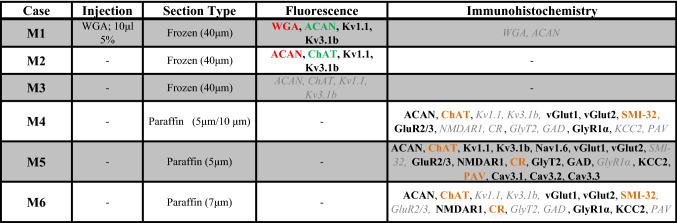
Summary of experimental protocols, histological procedures and immunohistochemical details for tissue obtained from six macaque monkeys (cases M1–M6)

Antibodies in gray italics denote histochemical tests that were not illustrated in the figures. Antibodies in bold denote IHC labeling shown in the figures. Colors of antibodies match the visualized chromogen of a particular antibody staining

*IF* immunofluorescence; *IHC* immunohistochemistry (peroxidase)

The *Macaca mulatta* specimen (M1) received an injection with the tract-tracer wheatgerm agglutinin (WGA; 10 μL 5%; EY Laboratories) into the myotendinous junction of the lateral rectus muscle of one eye to retrogradely label MIF motoneurons. After three days of survival, the animal was sacrificed with an overdose of pentobarbital (90 mg/kg body weight) and transcardially perfused with 0.9% saline followed by 2–3 L of 4% PFA in 0.1 M PB (pH 7.4). The extracted brainstem of this specimen, as well as two additional *Macaca nemestrina* specimens (M2 and M3), were similarly prepared for sectioning on a freezing microtome by immersion in increasing concentrations of 10–30% sucrose dissolved in 0.1 M PB solution. Frozen brainstems were cut with a Leica cryostat at 40 μm thickness in the transverse plane, and used for immunofluorescence staining.

Brainstems from three additional *Macaca mulatta* cases from previous projects, which were embedded in paraffin, were also included in the analysis (Zeeh et al. 2020). Thin serial sections (5 µm (M4, M5), 7 µm (M6), 10 µm (M4)) were cut and immunostained after deparaffination, rehydration and antigen retrieval protocols. In brief, antigen retrieval was accomplished by rinsing the rehydrated sections in distilled water and reacting in 0.01 M sodium citrate buffer (pH 6.0) at 1160 W power in a microwave (AEG, Micromat) three times for 3 min, each. After cooling to room temperature, sections were transferred to a Tris buffered saline (TBS; pH 7.4) for subsequent immunoperoxidase-based staining.

All experimental procedures conformed to the State and University Regulations on Laboratory Animal Care, including the Principles of Laboratory Animal Care (NIH Publication 85-23, Revised 1985), and were approved by the Animal Care Officers and Institutional Animal Care and Use Committees at the University of Washington, where all surgical interventions were made.

### Antisera

#### Choline acetyltransferase (ChAT)

Cholinergic motoneurons were detected with an affinity-purified polyclonal goat anti-ChAT antibody (Cat #: AB144P; RRID: AB_2079751; Chemicon, Temecula, CA, USA) directed against the whole enzyme isolated from the human placenta, which is identical to the enzyme expressed in the brain (Bruce et al. 1985). This antibody recognizes a 68–70 kDa protein. The appearance and distribution of ChAT-immunopositive neurons identified with this antibody in the present study comply with the respective results of previous studies (Eberhorn et al. 2005; Horn et al. 2018). A 1:75 dilution was used for the immunoperoxidase-based method and a 1:25 dilution for the immunofluorescence-based detection.

#### Wheat germ agglutinin (WGA)

The tracer wheat germ agglutinin (WGA; EY Labs, San Mateo, CA, USA) was detected with a polyclonal goat antibody (Cat #: SM1353AS-2024; RRID: AB_2315611; Vector, Burlingame, CA, USA). A 1:5000 dilution was used for the immunofluorescence-based detection.

#### Aggrecan (ACAN)

Perineuronal nets were detected with the monoclonal mouse anti-aggrecan antibody (Cat #: SM1353; RRID: AB_972582; Acris Antibodies GmbH, Herford, Germany), which was developed to identify human aggrecan protein, a proteoglycan component of the cartilage matrix. A 1:75 dilution was used for the immunoperoxidase-based method and a 1:25 dilution for the immunofluorescence-based detection.

#### Voltage-gated potassium channel subunits Kv1.1 and Kv3.1b

The voltage-gated potassium channel subfamily A member 1 (KCNA1) subunit was detected with a polyclonal rabbit antibody (Cat #: APC-009; RRID: AB_2040144; Alomone Labs, Jerusalem, ISRAEL). This antibody recognizes the intracellular Kv1.1 C-Terminus epitope, corresponding to amino acid residues 416–495 of the mouse (*Mus musculus*) Kv1.1 protein. In this study, a 1:750 dilution was used for the immunoperoxidase-based method and a 1:250 dilution for the immunofluorescence-based detection.

The antibody against Kv3.1b amino acid residues 567–585, corresponding to the C-terminus of the voltage-gated potassium channel subunit KCNC1 (RRID: AB_2040166) was raised in rabbit (Weiser et al. 1995). In this study, a 1:6000 dilution was used for the immunoperoxidase-based method and a 1:2000 dilution for the immunofluorescence-based method.

#### Voltage-gated sodium channel subunit Nav1.6

The voltage-gated sodium channel type VIII alpha subunit (SCNA8) was detected with a polyclonal rabbit antibody (Cat #: ASC-009; RRID: AB_2040202; Alomone Labs, Jerusalem, ISRAEL). This antibody recognizes amino acid residues 1042–1061 of the rat Nav1.6 peptide. In this study, a 1:500 dilution was used.

#### Low-voltage activated calcium channel subunits (Cav3.1, Cav3.2, Cav3.3)

Voltage-dependent T-type calcium channel subunits Cav3.1 (CACNA1G, α1G; Cat #: ACC-021; RRID: AB_2039779), Cav3.2 (CACNA1H, α1H; Cat #: ACC-025; RRID: AB_2039781) and Cav3.3 (CACNA1I, α1H; Cat #: ACC-009; RRID: AB_2039783) were detected with polyclonal rabbit antibodies from (Alomone Labs, Jerusalem, ISRAEL). The Cav3.1 antibody recognizes intracellular amino acid residues 1–22 of the rat CACNA1G at the N-terminus. The Cav3.2 antibody recognizes amino acid residues 581–595 of the rat CACNA1H at the intracellular loop between domains D1 and D2. The Cav3.3 antibody recognizes amino acid residues 1053–1067 of the rat Cav3.3 between the intracellular domains II and III. In this study, a 1:1000 dilution was used for all three antibodies.

#### AMPA receptor GluR2/3

Glutamate receptors (GluR) 2 and 3 were detected with a polyclonal rabbit antibody (Cat #: AB1506; RRID: AB_90710; Chemicon, Temecula, CA, USA), which recognizes the C-terminus (EGYNVYGIESVKI) of the rat GluR2 peptide, which is nearly identical with the C-terminus of GluR3. Here, a 1:500 dilution was used.

#### NMDA receptor 1

The NMDA receptor 1 (*N*-methyl-d-aspartate receptor channel, subunit zeta-1) was detected with a monoclonal mouse antibody (Cat #: MAB363; RRID: AB_94946; Chemicon, Temecula, CA, USA), which recognizes amino acid residues 660–811 located in the extracellular loop between transmembrane regions III and IV of the NMDAR1. In this study, a 1:1000 dilution was used.

#### Glutamate decarboxylase (GAD65/67) and glycine transporter 2 (GlyT2)

GABAergic synaptic terminals were detected by a polyclonal rabbit anti-glutamate decarboxylase 65 and 67 (GAD65/67) antibody (Cat #: AB1511; RRID; AB_90715; Chemicon, Temecula, CA, USA), which recognizes C-terminus residues 572–585. GAD65 is membrane-anchored (585 a.a.) and is responsible for vesicular GABA production, whereas GAD67 is located in the cytoplasm (594 a.a.) and is responsible for a significant cytoplasmic GABA production. A 1:2000 dilution was used.

Glycinergic synaptic terminals were detected by a polyclonal sheep antibody (Cat #: AB1771; RRID: AB_90945; Chemicon, Temecula, CA, USA), which recognizes a synthetic peptide from the C-terminus of the glycine transporter 2 as predicted from the cloned rat GlyT2. A 1:5000 dilution was used.

#### Glycine receptor 1α

Glycine receptor 1α was detected by a monoclonal (clone mAb4a) mouse antibody (Cat #: 146 011; RRID: AB_887722; Synaptic Systems, Göttingen, Germany), which recognizes amino acid residues 96–105 from the rat glycine receptor α1. A 1:300 dilution was used.

#### K^+^/Cl^−^ cotransporter (KCC2)

The potassium chloride symporter 2 (KCC2) was detected by a polyclonal rabbit antibody (Cat #: 07-432; RRID: AB_310611; Chemicon, Temecula, CA, USA), which recognizes amino acid residues 932–1043 of the rat KCC2 at the N-terminus. A 1:4000 dilution was used.

#### Calretinin (CR) and parvalbumin (PAV)

The calcium-binding protein calretinin (CR) was detected with a polyclonal rabbit antibody (Cat #: 7699/3H; RRID: AB_10000321; Swant, Marly, Fribourg, Switzerland) as described previously (Fairless et al. 2019). The calcium-binding protein parvalbumin (PAV) was detected with a monoclonal mouse antibody (Cat #: 235; RRID: AB_10000343; Swant, Marly, Fribourg, Switzerland). In this study, a 1:2500 dilution was used for both antibodies.

#### Vesicular glutamate transporters (vGlut1 and vGlut2)

The vesicular glutamate transporter 1 (vGlut1/ SLC17A7) was detected with a polyclonal rabbit antibody (Cat #: 135 303; RRID: AB_887875; Synaptic Systems, Göttingen, Germany). The vesicular glutamate transporter 2 (vGlut2/SLC17A6) was detected with a monoclonal mouse antibody (Cat #: MAB5504; RRID: AB_2187552; Chemicon, Temecula, CA, USA). Both, vGlut1 and vGlut2 mediate the uptake of glutamate into synaptic vesicles at the presynaptic nerve terminals of excitatory neurons, and usually show complementary expression patterns (Fremeau et al. 2004). In this study, a 1:3000 dilution for vGlut1 and a 1:4000 dilution for vGlut2 were used.

The specificities of all antibodies were validated with the first antibody omission control and pre-absorption control tests.

### Staining methods

#### Combined immunofluorescence labeling of tracer-stained motoneurons

Transverse sections through the pontomedullary junction were processed for different combinations of immunofluorescence staining. For simultaneous detection of WGA and PNs, floating sections of case M1 were incubated in 5% normal donkey serum in 0.1 M Tris-buffered saline (TBS; pH 7.4), containing 0.3% Triton X-100 (NDS-TBS-T) for 1 h at room temperature. Subsequently, the sections were processed with a mixture of mouse anti-ACAN (1:25), goat anti-WGA (1:250) and optionally one of the voltage-gated potassium channel markers (rabbit anti-Kv1.1, 1:250 or rabbit anti-Kv3.1b, 1:2000) in NDS-TBS-T for 48 h at 4 °C. After washing three times in 0.1 M TBS, the sections were treated with a mixture of Cy3-conjugated donkey anti-rabbit IgG (1:200; Dianova), Alexa Fluor 488-tagged donkey anti-mouse IgG (1:200; Molecular Probes, Eugene, OR, USA) and DyLight 512 tagged donkey anti-goat IgG (1:100, Dianova) for 2 h at room temperature. After a short rinse in distilled water, sections were dried and coverslipped with DPX mounting medium (Gel/Mount; Biomeda, San Francisco, CA, USA) and stored in darkness at 4 °C.

#### Single and double immunoperoxidase stainings of consecutive paraffin sections

For single immunoperoxidase staining, paraffin-embedded brainstem sections of cases M4, M5 and M6 were washed in 0.1 M TBS (pH 7.4) and treated with 1% H_2_O_2_ in TBS for 30 min to block endogenous peroxidase activity subsequent to deparaffination, rehydration and antigen retrieval protocols. Sections were then processed with a primary antibody of choice (see Methods, Table 2) in 0.1 M TBS (pH 7.4), containing 0.3% Triton X-100 (NDS-TBS-T) in humid chambers for 48 h at 4 °C*.* Subsequent to primary antibody incubation, all markers were visualized by the binding of biotinylated secondary antibodies (1:200; Vector Lab) followed by extravidin-peroxidase (1:1000; Sigma) and diaminobenzidine (DAB) as a chromogen to yield a brown colored, or DAB-Nickel as chromogen to yield a black colored precipitate.

**Table 2:** Summary of primary antibodies and dilutions for the immunolabeling

Antibody	Host	Antigen	Manufacturer	Antibody registry number (RRID)	Dilution
WGA	Goat/polyclonal	Wheat germ agglutinin	EY Labs, San Mateo,CA, USA	AB_2315611	1:250 (IF)
ACAN	Mouse/monoclonal	Aggrecan	Acris Antibodies GmbH, 32052 Herford, Germany	AB_972582	1:25 (IF), 1:75 (IHC)
ChAT	Goat/polyclonal	Choline acetyltransferase	Chemicon, Temecula, CA, USA	AB_2079751	1:25 (IF), 1:50 (IHC)
Kv1.1	Rabbit/polyclonal	Voltage-gated potassium channel 1.1	Alomone Labs Jerusalem BioPark (JBP)	AB_2040144	1:250 (IF), 1:750 (IHC)
Kv3.1b	Rabbit/polyclonal	Voltage-gated potassium channel 3.1b	(Weiser, Bueno et al. 1995)	Härtig (AB_2040166)	1:2000 (IF), 1:6000 (IHC)
Nav1.6	Rabbit/polyclonal	Voltage-gated sodium channel 1.6	Alomone Labs Jerusalem BioPark (JBP)	AB_2040202	1:500 (IHC)
vGlut1	Rabbit/polyclonal	Vesicular glutamate transporter 1	Synaptic Systems, Göttingen, Germany	AB_887875	1:3000 (IHC)
vGlut2	Mouse/monoclonal	Vesicular glutamate transporter 2	Chemicon, Temecula, CA, USA	AB_2187552	1:4000 (IHC)
SMI-32	Mouse/monoclonal	Nonphosphorylated neurofilament marker H	SM1353, Acris Antibodies	AB_2715852	1:2500 (IHC)
GluR2/3	Rabbit/polyclonal	Glutamate (AMPA) receptor 2/3	Chemicon, Temecula, CA, USA	AB_90710	1:500 (IHC)
NMDAR1	Mouse/monoclonal	(NMDA) receptor 1	Chemicon, Temecula, CA, USA	AB_94946	1:1000 (IHC)
CR	Rabbit/polyclonal	Calretinin	Swant, Marly, Fribourg, Switzerland	AB_10000321	1:2500 (IHC)
GlyT2	Sheep/polyclonal	Glycine transporter 2	Chemicon, Temecula, CA, USA	AB_90945	1:5000 (IHC)
GAD	Rabbit/polyclonal	Glutamate decarboxylase 65 and 67	Chemicon, Temecula, CA, USA	AB_90715	1:2000 (IHC)
GlyR1α	Mouse/monoclonal	Glycine receptor 1α	Synaptic Systems, Göttingen, Germany	AB_887722	1:300 (IHC)
KCC2	Rabbit/polyclonal	Potassium-chloride cotransporter 2	Chemicon, Temecula, CA, USA	AB_310611	1:4000 (IHC)
Cav3.1	Rabbit/polyclonal	T-type voltage-gated calcium channel 3.1	Alomone Labs Jerusalem BioPark (JBP)	AB_2039779	1:1000 (IHC)
Cav3.2	Rabbit/polyclonal	T-type voltage-gated calcium channel 3.2	Alomone Labs Jerusalem BioPark (JBP)	AB_2039781	1:1000 (IHC)
Cav3.3	Rabbit/polyclonal	T-type voltage-gated calcium channel 3.3	Alomone Labs Jerusalem BioPark (JBP)	AB_2039783	1:1000 (IHC)
PAV	Mouse/monoclonal	Parvalbumin	Swant, Marly, Fribourg, Switzerland	AB_10000343	1:2500 (IHC)

Series of paraffin sections from cases M4, M5 and M6 were processed for concomitant detection of ChAT-immunopositive motoneurons and ACAN-containing PNs as described previously (Horn et al. 2018). Combined detection of two primary antibodies was carried out similarly to the single staining in a sequential manner, where the first antigen was detected by the reaction with DAB-Ni yielding a black precipitate. Subsequently, sections were treated with 1% H_2_O_2_ in TBS for 30 min as the first step of the second round of staining with the same serum. In these cases, the second antigen was detected with a simple DAB reaction protocol yielding a brown precipitate.

For preservation and scanning, sections were extensively washed with TBS (pH 7.4), briefly rinsed in distilled water, air-dried and cover-slipped with DPX mounting medium (Sigma-Aldrich, Steinheim, Germany).

### Analysis of stained sections

Sections containing fluorescent labeling were examined with a Leica microscope DMRB (Bensheim, Germany) equipped with appropriate filters for red fluorescent Cy3 (N2.1), green fluorescent Alexa 488 (I3), or blue fluorescence imaging capability. Images from selected preparations were captured with a laser-scanning confocal microscope (Leica SP5, Mannheim, Germany) at 10× or 63× magnification. Triple imaging for Dylight, Alexa 488 and Cy3 fluorophores were sequentially performed at 405, 488 or 543 nm excitation wavelength, respectively. Z-stack series were collected every 0.5 μm (at 63×) or 2 μm (at 10×) for each section. Image stacks were processed with Fiji/ImageJ software (https://imagej.net/Fiji, SCR_003070). Contrast and brightness of the final composite images were adjusted to reflect the appearance of the labeling, as seen through the microscope using Fiji software.

Brightfield images of paraffin-embedded sections were captured either with a digital camera (Microfire; Optronics, USA) using PictureFrame 2.2 software (Optronics, USA) or with a slide scanner (Mirax MIDI, Zeiss), equipped with a Plan-Apochromat objective (Zeiss, 20×). The digitized images were viewed and captured with the free software Panoramic Viewer (3DHistech; 1.152.3) and Case Viewer (3DHistech; v2.2). Corresponding detailed views of equally arranged and magnified images of adjacent sections were analyzed on the computer screen. The same neurons were identified by their location with the help of anatomical landmarks, such as blood vessels.

### Quantification of immunopositive puncta and statistical analysis

For quantification, images were captured with a slide scanner (Mirax MIDI, Zeiss), equipped with a Plan-Apochromat objective (Zeiss, 20×), loaded into Fiji software, followed by a conversion into RGB format and sharpening for better edge detection. Neurons to be investigated were identified using consecutive PN/ChAT-stained sections. Somatic perimeters of labeled neurons in the adjacent sections were measured using manual selection with the freehand tool after setting the corresponding scale. Finally, immunopositive puncta were manually counted, and number of puncta per µm perimeter were calculated by dividing the number of puncta by the measured somatic perimeter for each neuron. Quantification of somatic versus dendritic glutamatergic inputs was performed on a 10 µm thick section from case M4 stained for vGlut2 and ChAT antibodies. Only dendrites in continuation with the soma were included in the analysis. vGlut2-immunopositive puncta were counted along a hand-drawn line of the perimeters and the associated dendrite(s). For comparative quantification of transmitter inputs to the different neuronal populations in nVI and nIV, the density of vGlut2-, GlyT2- and GAD65/67-immunopositive puncta were counted along the somatic perimeters in two to three 5 µm thick sections at different levels of the respective nuclei of two cases.

All data sets showed a normal distribution according to the Kolmogorov–Smirnov Test of Normality, which is a prerequisite for subsequent *t-*Test analyses of different sized samples. Two-tailed Student’s *t-*Test for two independent means was performed for each comparison (i.e., MIF versus SIF motoneurons, or dendritic versus somatic locations) to assess differences in mean numbers of synaptic terminals (puncta/µm) between populations.

## Results

### MIF and SIF motoneurons differ in voltage-gated potassium channel profiles

#### Differential Kv1.1 and Kv3.1b channel distribution in neurons of the abducens and trochlear nuclei

**Fig. 1:**
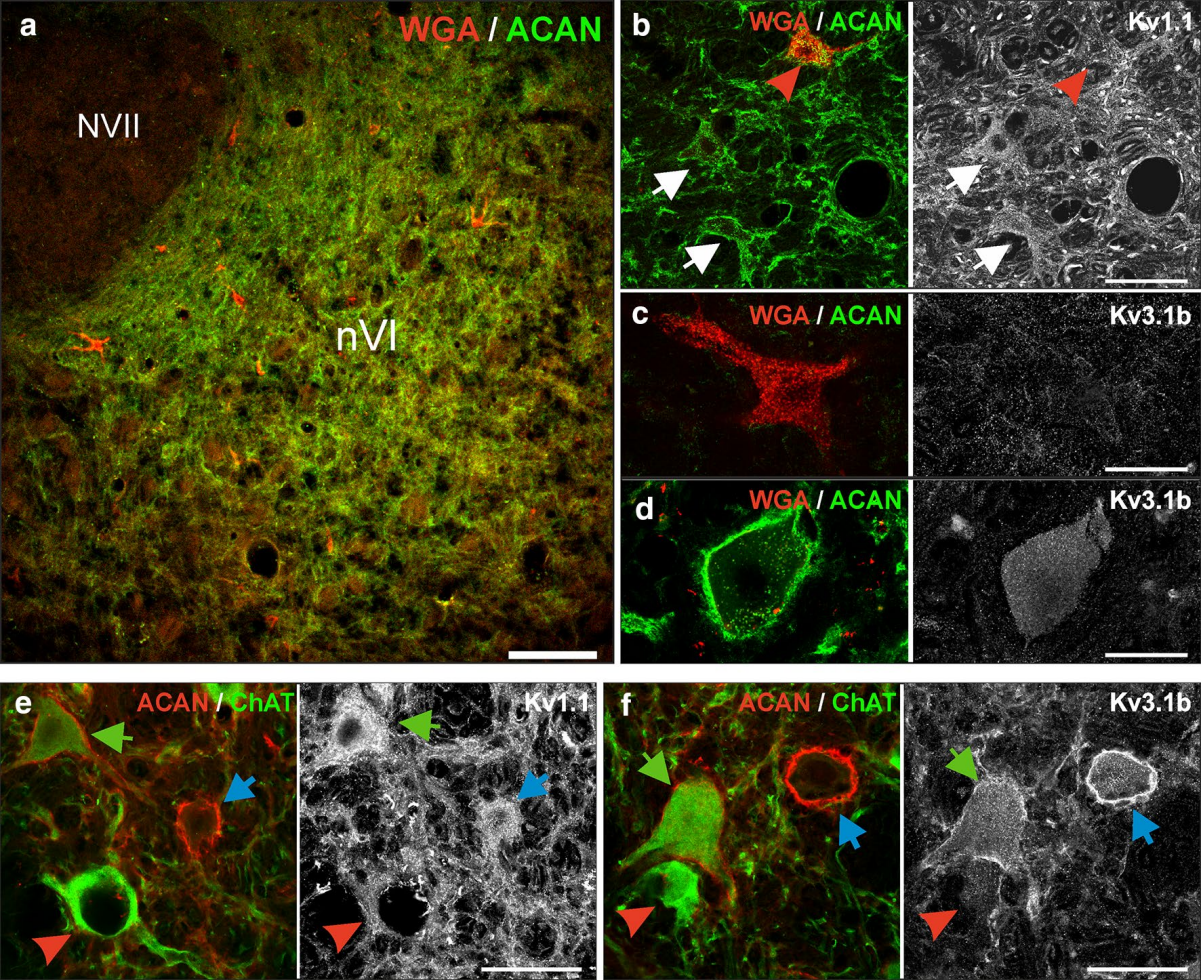
Immunofluorescent detection of Kv1.1 and Kv3.1b in the abducens nucleus. **a** Transverse section through the abducens nucleus (nVI) depicting retrogradely labeled motoneurons (MNs) of multiply-innervated muscle fibers (MIF) (red) after injection of wheat germ agglutinin (WGA) into the myotendinous junction of the lateral rectus muscle. These cells lack aggrecan (ACAN)-based perineuronal nets (PN) (green). **b** Detailed view of a different section (left) demonstrating WGA-labeled MIF MNs (red arrowhead) and PN-bearing SIF MNs or internuclear neurons (INT) (white arrows) both of which lacked WGA labeling; SIF MNs or INTs (right) express strong immunoreactivity for Kv1.1 (white arrows), whereas MIF MNs exhibit a weak Kv1.1 signal (red arrowhead). **c** Close-up of a WGA-labeled MIF MN (left) lacking Kv3.1b-immunoreactivity (right). **d** Close-up of a PN-immunopositive SIF MN or INT (left) with strong Kv3.1b-immunoreactivity (right). **e, f** Close-up examples of abducens neurons following triple immunofluorescence staining for ACAN (red), choline acetyltransferase (ChAT) (green) and Kv1.1 (**e**, white) or Kv3.1b (**f**, white) in two different cases; note in **e** the strong Kv1.1-immunoreactivity in SIF MNs (green arrows) and INTs (blue arrows) visible as somatic staining around the cell nucleus. By comparison, there are low levels of Kv1.1-immunoreactivity in MIF MNs (red arrowhead); SIF MNs (green arrows) and INTs (blue arrows) in **f** express very strong Kv3.1b-immunoreactivity, which is absent in MIF MNs (red arrowheads). NVII, facial nerve. Scale bar represents 200 μm in **a**, 50 μm in **b** and 30 μm in **c**–**f**

As previously shown, WGA-injection into the myotendinous junction of the lateral rectus muscle (case M1) resulted in retrogradely labeled, small to medium-sized MIF motoneurons mainly in the periphery of the abducens nucleus (Büttner-Ennever et al. 2001). These neurons lack aggrecan (ACAN)-based perineuronal nets (PN) in contrast to SIF motoneurons and INTs (Eberhorn et al. 2005) (Table 1; Fig. 1a–c). Combined immunofluorescence detection of WGA and ACAN, as well as selected Kv channels, revealed a weak labeling of Kv1.1 ion channels in MIF motoneurons, (Fig. 1b, red arrowhead), while ACAN-immunopositive SIF motoneurons or INTs, which were not labeled by tracer injections into the myotendinous junction of the muscle, showed a strong Kv1.1 signal within their soma (Fig. 1b, white arrows). Kv3.1b-immunoreactivity was absent in retrogradely labeled MIF motoneurons (Fig. 1c) but was present in all ACAN-immunopositive neurons (Fig. 1d). In additional cases (M2, M3; Table 1), a second approach using triple immunofluorescence for Kv channels, ACAN and choline acetyltransferase (ChAT) was applied, which allowed a distinction between SIF motoneurons and INTs (Fig. 1e, f). SIF motoneurons, defined by ChAT- and PN-immunopositivity, exhibited intense Kv1.1- and Kv3.1b-immunofluorescence (Fig. 1e, f, green arrows). Kv1.1-immunolabeling appeared as strongly stained clusters within the cytoplasm only sparing the nucleus, while the signal along the membrane was weak (Fig. 1e; green arrow). In contrast, the Kv3.1b labeling was strongest along the membranes of the soma and proximal dendrites, while weak immunoreactivity was observed within the somatic cytoplasm (Fig. 1f, green arrow). As reported above, ChAT-immunopositive but ACAN-immunonegative MIF motoneurons showed no Kv3.1b-immunoreactivity (Fig. 1f, red arrowhead). In addition, the low level of Kv1.1-immunopositivity did not present as clustered signals around the cell nucleus and was barely detectable along the somatic membrane (Fig. 1e, red arrowhead). Finally, ChAT-immunonegative INTs, which were densely ensheathed by PNs, were strongly labeled by antibodies directed against both Kv1.1 and Kv3.1b (Fig. 1e,f, blue arrows). In fact, INTs stood out within the abducens nucleus by their prominent PN and Kv3.1b-immunolabeling (Fig. 1e, f, blue arrows).

**Fig. 2:**
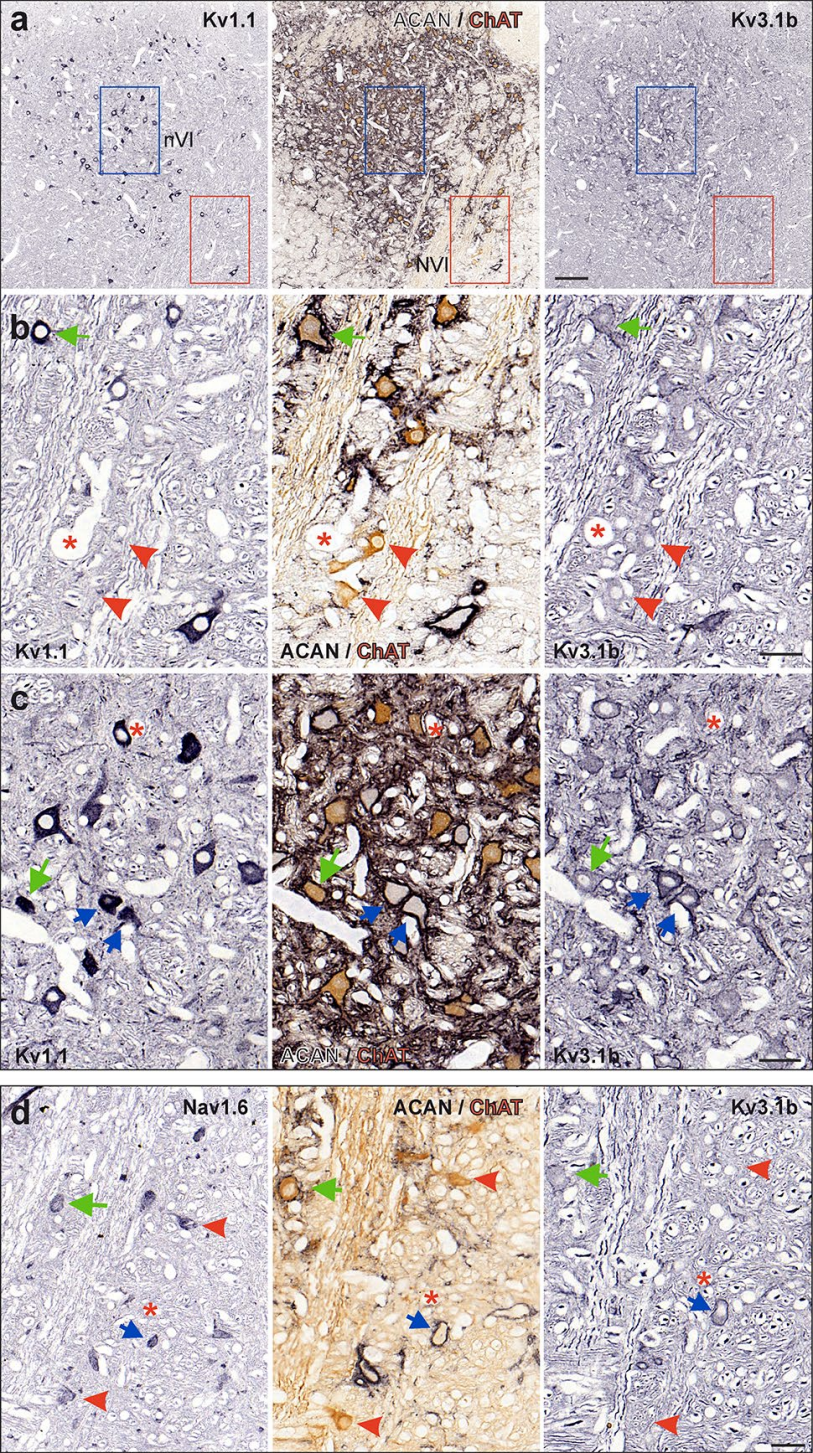
Immunoperoxidase labeling of Kv1.1, Kv3.1b and Nav1.6 proteins in the abducens nucleus. **a** Consecutive coronal paraffin sections through the abducens nucleus (nVI) immunostained for Kv1.1 (left), Kv3.1b (right), combined aggrecan (ACAN)-based perineuronal nets (PN; black) and choline acetyltransferase (ChAT; brown, middle), respectively. Note the regional variability in the intensity of Kv1.1-immunolabeling within the nucleus; colored boxes indicate the areas illustrated at higher magnification in b (red) and c (blue), respectively. **b** Close-up comparing Kv1.1 and Kv3.1b expression in motoneurons (MNs) of multiply-innervated muscle fibers (MIF) (red arrowheads) and singly-innervated muscle fibers (SIF) (green arrow). **c** Close-up of Kv1.1 and Kv3.1b expression in internuclear neurons (INTs) (blue arrows) and SIF MNs (green arrow). **d** Close up of Nav1.6-immunolabeling related to Kv3.1b expression in three abducens neuronal populations (SIF MNs: green arrow; MIF MNs: red arrowheads; INTs: blue arrow). Scale bar represents 200 μm in **a**, and 50 μm in **b**–**d**

**Fig. 3:**
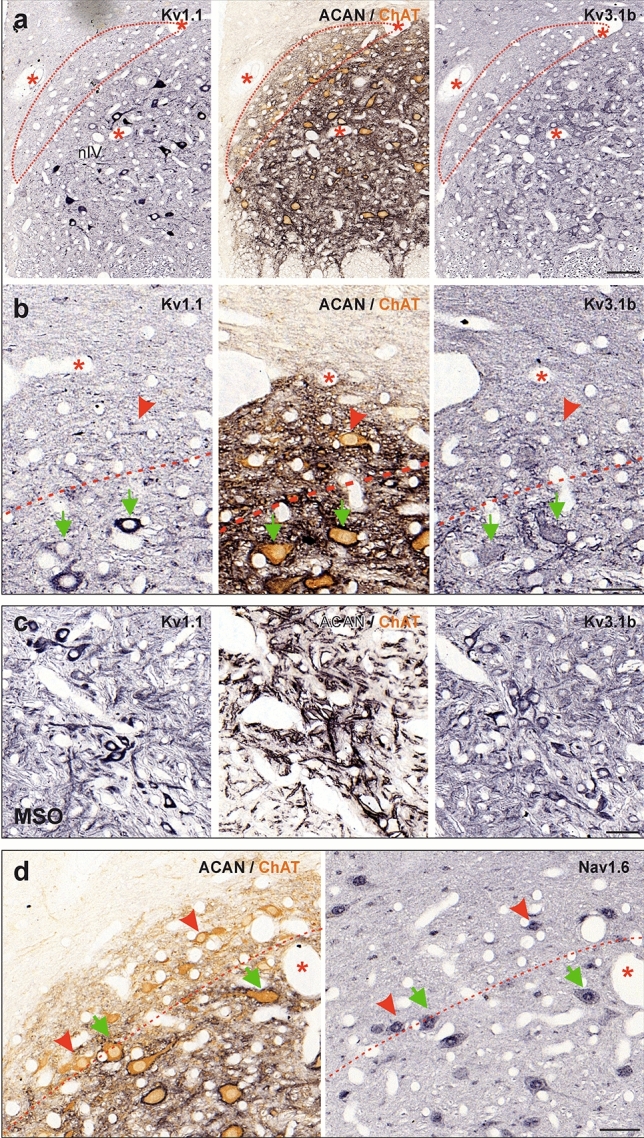
Immunoperoxidase labeling of Kv1.1, Kv3.1b and Nav1.6 proteins in the trochlear nucleus. **a, b** Consecutive coronal paraffin sections depicting an overview (**a**) and close-up from a different section (**b**) through the trochlear nucleus (nIV) immunostained for Kv1.1 (left), Kv3.1b (right), combined aggrecan (ACAN)-based perineuronal nets (PN; black) and choline acetyltransferase (ChAT; brown, middle), respectively; the red dashed lines delineate the dorsal cap of nIV containing motoneurons of multiple-innervated muscle fibers (MIF MN); note the regional variability in the intensity of Kv1.1-immunolabeling within the nucleus. **c** Medial superior olivary (MSO) neurons adjacent to the abducens nucleus on the same section as a positive control for the immunohistochemical specificity of the antibody staining. **d** Close-up of Nav1.6-immunolabeling in MIF (red arrowheads) and SIF MNs (green arrows) in nIV. Red dashed lines indicate the tentative boundary between SIF and MIF MNs. Scale bar represents 100 μm in **a** and 50 μm in **b**–**d**

To confirm the lack of Kv3.1 expression in MIF motoneurons, the general presence of Kv channels was validated by the more sensitive immunoperoxidase staining using selected sets of three consecutive 5 or 7 μm paraffin sections through the abducens and trochlear nuclei in three additional cases (Figs. 2, 3; Case M4, M5, M6; see Table 1). With this method, Kv1.1- and Kv3.1b-immunoreactivity was detected as black DAB-Ni precipitate (Figs. 2a–c, 3a–c, left and right columns) on sections adjacent to the one immunostained for the PN marker ACAN (black) and the motoneuron marker ChAT (brown; Figs. 2a–c, 3a–c, middle columns). Using ACAN/ChAT-immunostaining, MIF MNs were identified by the positive ChAT, but negative ACAN labeling (red arrowheads), SIF MNs were identified by positive ChAT and ACAN labeling (green arrows), and INTs in the abducens nucleus were identified by negative ChAT, but positive ACAN labeling (Fig. 2c, blue arrows) for evaluation of the same neurons on adjacent sections. Varying intensities of somatic immunoreactivity for Kv1.1 were detected in both the abducens and trochlear nuclei (Figs. 2a, b, 3a, b, left columns), whereas the Kv3.1b-immunolabeling was generally uniform in the cytoplasm, with a predominant localization along the somatic and proximal dendritic membrane (Figs. 2a,b, 3a, b, right columns). Unlike scattered abducens MIF motoneurons, trochlear MIF motoneurons are clustered in a dorsal cap facilitating identification by the lack of ACAN-immunopositive PNs (Fig. 3a, red dashed line boundaries). In both motor nuclei, the ChAT-immunopositive PN-ensheathed SIF motoneurons showed strong Kv3.1b-immunostaining, a feature that was clearly absent from MIF motoneurons (Figs. 2b, 3b, middle and right columns, red arrowheads). Kv1.1-immunolabeling was equally strong in SIF motoneurons, but only weakly expressed in MIF motoneurons (Figs. 2b,c, 3b, left columns, green arrows and red arrowheads, respectively). As demonstrated by immunofluorescence staining (see above), ChAT-immunonegative abducens INTs exhibited a very strong immunoreactivity for Kv3.1b and ACAN, which rendered them clearly visible even in the overview (Fig. 2a, c, middle and right columns; blue arrows). Their Kv1.1-immunolabeling was also strong and comparable to that of SIF motoneurons (Fig. 2a, c, left and middle columns, blue arrows). The specificity and localization of Kv1.1- and Kv3.1b-immunolabeling were qualitatively confirmed by visualization of the well-known expression pattern in medial superior olivary (MSO) neurons (Fig. 3c) (Mayadali et al. 2019; Nabel et al. 2019), where strong labeling was observed for both subunits, as well as for the PN marker ACAN, but not for ChAT, as expected for these neurons.

#### Nav1.6 subunit in neurons of the abducens and trochlear nuclei

The expression of the sodium channel subunit Nav1.6 is usually tightly correlated with the expression pattern of Kv3.1b subunits in agreement with a fast-spiking capacity for these neurons (Gu et al. 2018; Kodama et al. 2020). Therefore, expression of this sodium channel subunit was evaluated in SIF and MIF motoneurons in both, the abducens and trochlear nuclei (Figs. 2d, 3d, green arrows), and in abducens INTs (Fig. 2d, blue arrow). However, simultaneous expression of Kv3.1b and Nav1.6 was only found for SIF motoneurons and INTs, whereas only Nav1.6 expression was present in MIF motoneurons, which lacked Kv3.1b (Figs. 2d, 3d, red arrows).

### Excitatory transmitters and receptors in the abducens and trochlear nuclei

#### Glutamatergic synapses onto abducens and trochlear neurons utilize vGlut2, but not vGlut1

**Fig. 4:**
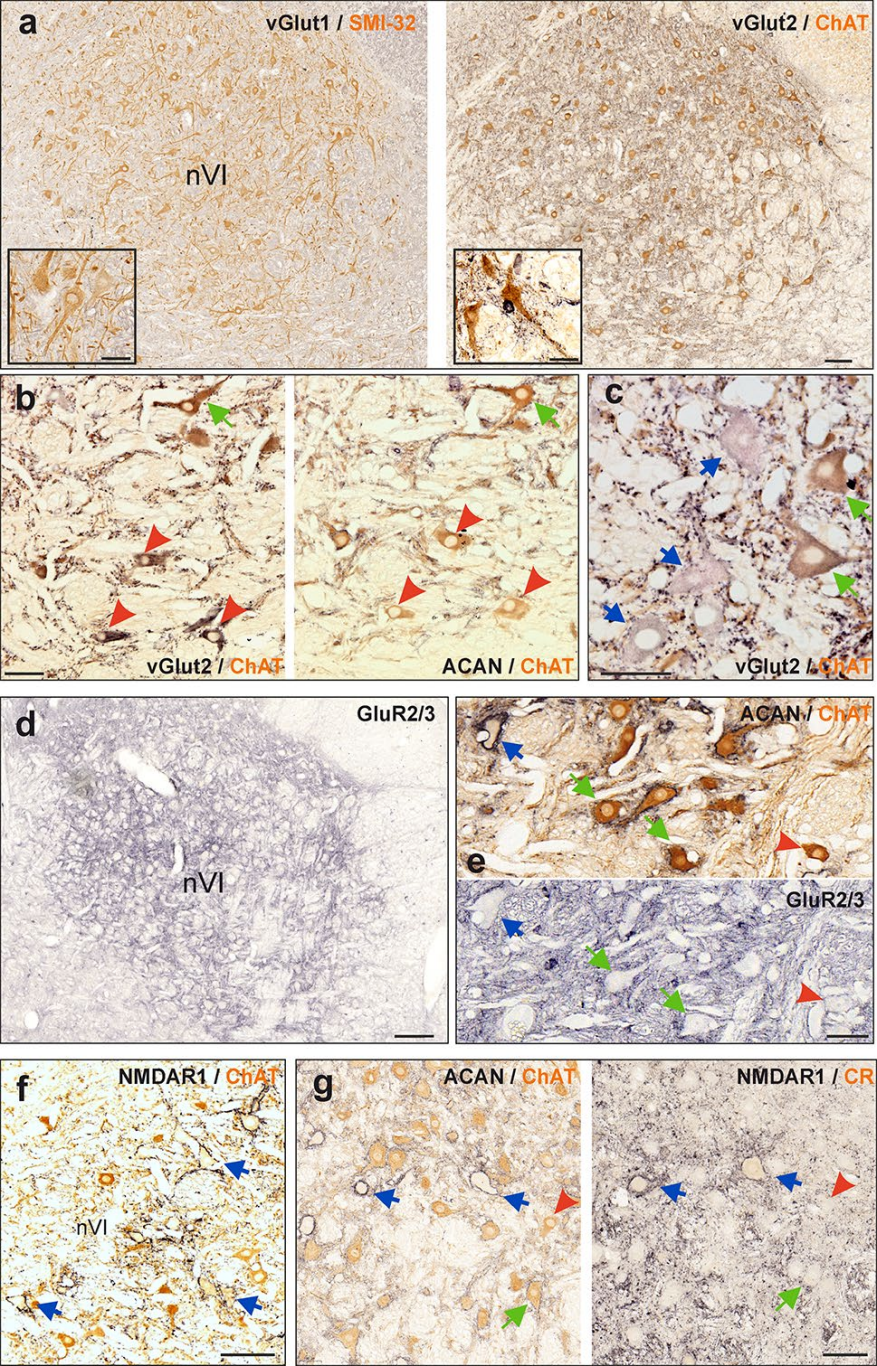
Immunohistochemistry of glutamatergic synapses onto abducens neurons. **a**–**g** Consecutive coronal paraffin sections illustrating glutamatergic presynaptic terminals (**a**–**c**) and postsynaptic receptors (**d**–**g**) of abducens (nVI) neurons identified by immunoperoxidase labeling (black). **a** Neurons in nVI immunostained for the vesicular glutamate transporter 1 (vGlut1) (black) and non-phosphorylated neurofilament with SMI32-antibody (brown; left), vGlut2 (black) and choline acetyltransferase (ChAT) (brown; right). Close-up examples of labeled neurons are illustrated in the insets in **a**. Close-up of vGlut2-positive terminals on MIF (**b**, red arrowheads) and SIF (**b, c,** green arrows) motoneurons (MNs) and internuclear neurons (INTs) (**c**, blue arrows) in nVI. **d** AMPA receptor GluR2/3-immunoreactivity in nVI. **e** Close-up of consecutive sections depicting GluR2/3-immunoreactivity of MIF (red arrowhead) and SIF (green arrows) MNs and INTs (blue arrow) in nVI. **f,g** NMDAR1-immunoreactivity in nVI; combined NMDAR1 and ChAT-immunostaining (brown) highlighting INTs with intense NMDAR1 labeling (**f**, blue arrows). Consecutive sections depicting ChAT-negative INTs (**g**, blue arrows) with weak calretinin (CR)-immunolabeling (**g**, right, brown) and strong somatic and dendritic NMDAR1 labeling; note that punctate labeling of NMDAR1 occurs on SIF MNs (**g**, green arrow), whereas MIF MNs exhibit no labeling (**g**, red arrowhead). Red arrowheads depict MIF MNs, green arrows depict SIF MNs and blue arrows depict INTs. Scale bar indicates 200 μm in **a, d**, 100 μm in **f** and 50 μm in **b, c, e, g** as well as for insets in **a**

**Fig. 5:**
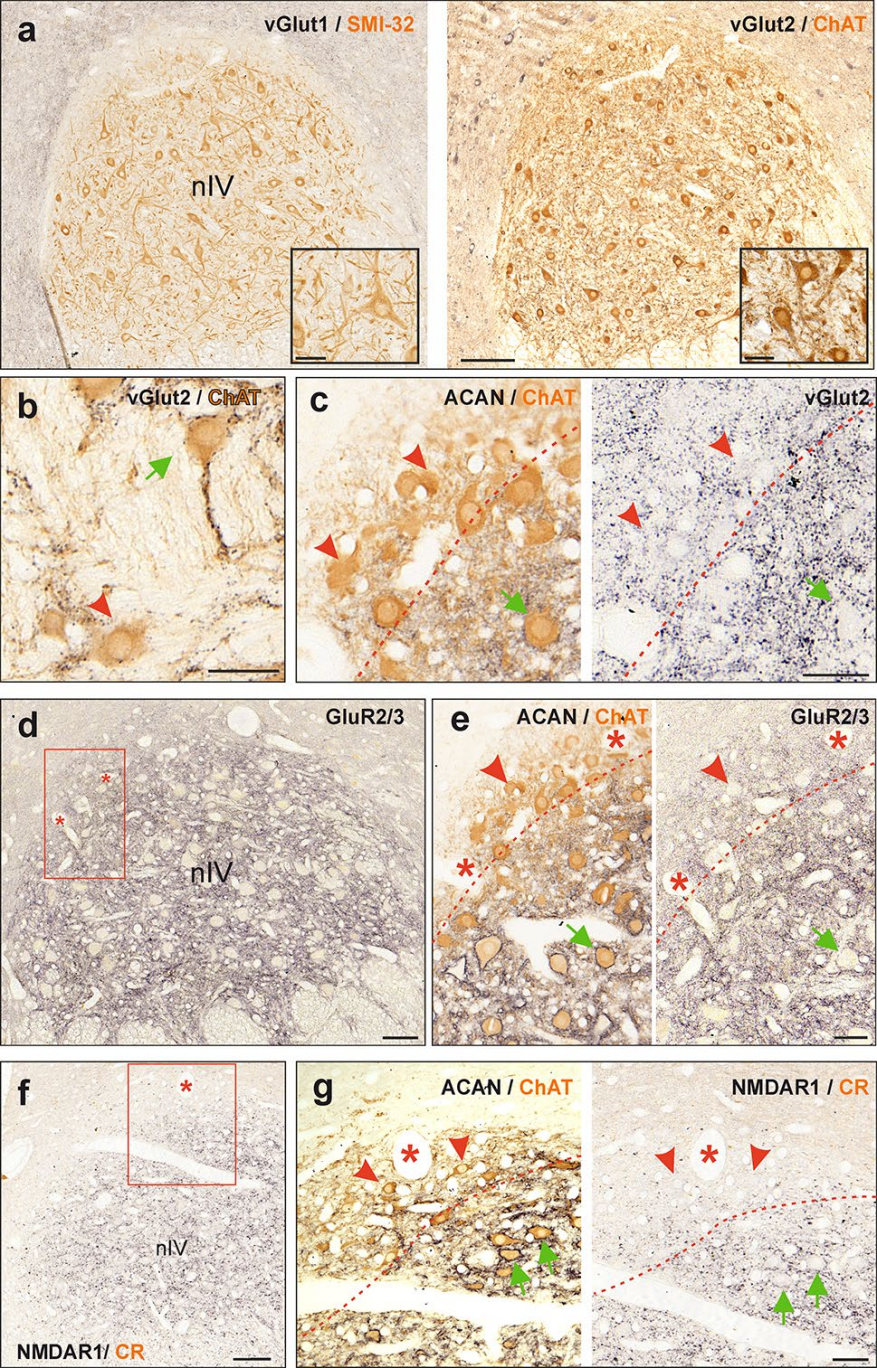
Immunohistochemistry of glutamatergic synapses onto trochlear neurons. **a**–**g** Consecutive coronal paraffin sections illustrating glutamatergic presynaptic terminals (**a**–**c**) and postsynaptic receptors (**d**–**g**) of trochlear (nIV) neurons identified by immunoperoxidase labeling (black). **a** Neurons in nIV immunostained for the vesicular glutamate transporter 1 (vGlut1) (black) and non-phosphorylated neurofilament with SMI32-antibody (brown) on the left, and for vGlut2 (black) and choline acetyltransferase (ChAT) (brown) on the right. Close-up examples are illustrated in the insets in **a**. **b** Example of somatic and dendritic distribution of vGlut2-immunopositive terminals on a SIF MN (green arrow) in nIV found on a 10 µm section. **c** Close-up of vGlut2-positive terminals on SIF (green arrow) within nIV and MIF MNs (red arrowheads) located within the dorsal cap of nIV. **d** AMPA receptor GluR2/3-immunoreactivity in nIV. **e** Close-up of the area outlined by the box in **d**, depicting weaker GluR2/3-immunolabeling in MIF MNs (red arrowhead) within the dorsal cap of nIV (right panel), as compared to SIF MNs (green arrow). **f** Combined NMDAR1- and CR-immunostaining in nIV. **g** Close-up of the area outlined by the box in **f** depicting NMDAR1 labeling only in SIF MNs (green arrows), but not in MIF MNs (red arrowheads) within the dorsal cap of nIV. Red arrowheads and green arrows depict MIF and SIF MNs, respectively. Red dashed lines indicate the tentative border delineating the dorsal cap of nIV. Scale bar indicates 200 μm in **a**, 100 μm in **d, f** and 50 μm in **b, c, e, g** as well as for insets in **a**

**Fig. 6:**
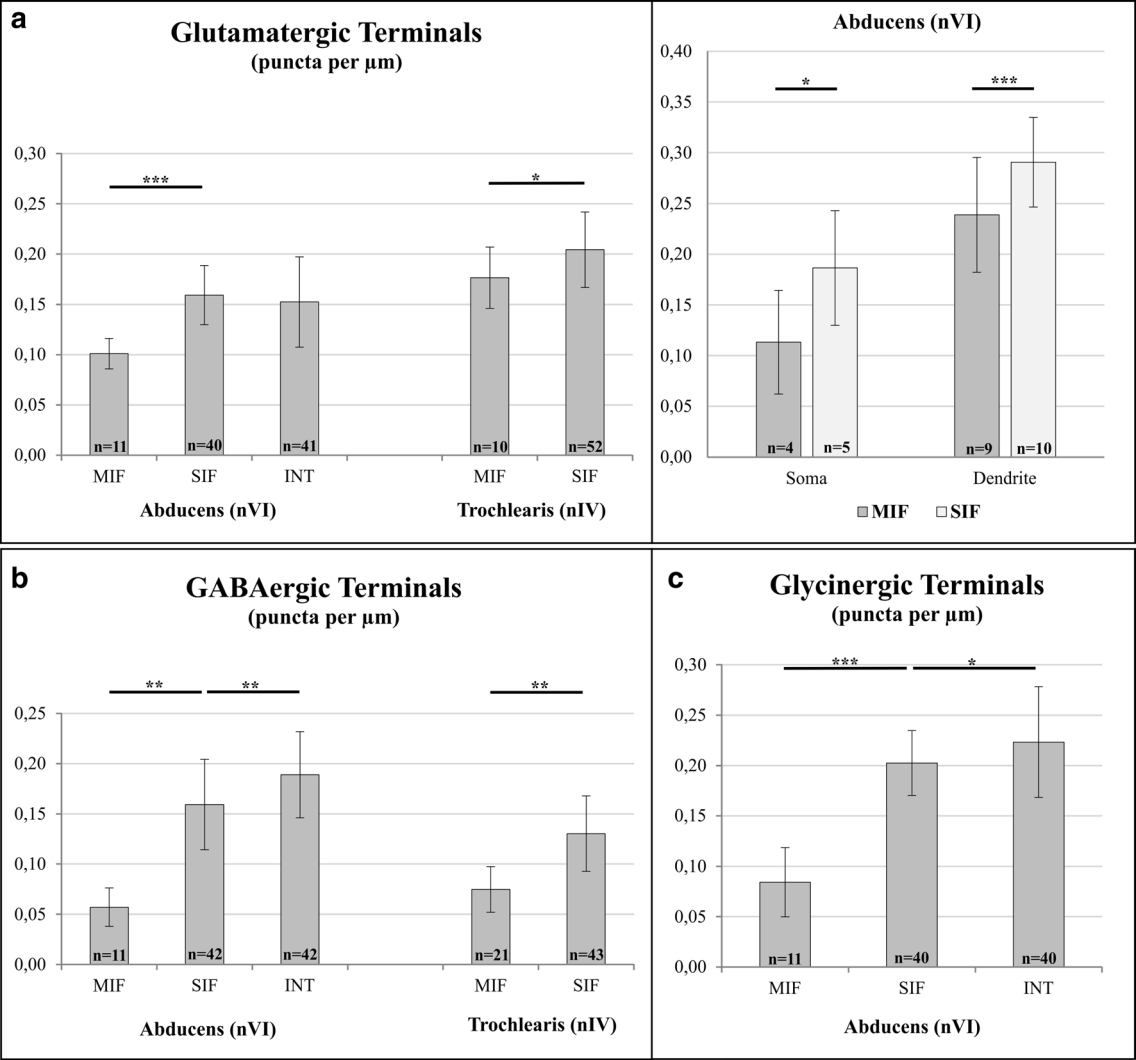
Quantification of vGlut2, GAD- and GlyT2-immunopositive puncta and statistical analysis. **a** Immunopositive glutamatergic terminals surrounding different types of neurons in the abducens (nVI) and trochlear (nIV) nuclei, quantified as numbers of puncta along the somatic membrane (puncta/µm) (left, obtained from 5 µm thick sections) and for comparison between dendritic and somatic locations (right, obtained from 10 μm thick section). **b, c** Immunopositive GABAergic (**b**) and glycinergic (**c**) synaptic structures quantified as number of puncta/µm along the somatic membrane of nVI and nIV neurons (obtained from 5 μm thick sections). Number (*n*) of analyzed neurons/measurements are indicated within the bars. Significant statistical differences between puncta/µm according to two-tailed *t*-test for two-independent means; *p < 0.05, **p < 0.01, ***p < 0.001

Glutamatergic inputs to cell groups in the motor nuclei were investigated on consecutive paraffin sections (Case M4, M5, M6) by immunostaining for the vesicular glutamate transporters 1 and 2 (vGlut1, vGlut2) known to be present in synaptic boutons (Fremeau et al. 2001). Combined immunostaining for either ChAT or non-phosphorylated neurofilament (SMI-32) were used as a marker for motoneurons (Figs. 4a, 5a, brown label). Numerous vGlut2-immunopositive puncta were present in both the abducens (Fig. 4a, right column) and trochlear nucleus (Fig. 5a, right column), most likely representing glutamatergic terminals. In contrast, no vGlut1-immunopositive puncta were found in either one of the two motor nuclei but occurred in abundance in adjacent areas, thus forming a sharp contrast that visually dissociated each nucleus clearly from the surrounding tissue (Figs. 4a, 5a, black label, left columns). While vGlut2-immunopositive puncta were observed along the somatic membrane of all neurons in the two nuclei, they were more concentrated on dendrites, as seen on thicker sections (7–10 μm; cases M4, M6), however, demonstrated in detail here only for the abducens nucleus (Fig. 5b). Accordingly, systematic quantification of the immunostaining in the abducens nucleus revealed that the mean density of vGlut2-immunopositive puncta was significantly higher along the dendritic, as compared to the somatic membrane, of both MIF (*p* < 0.01, *t*-test) and SIF (*p* < 0.001, *t*-test) motoneurons (Fig. 6a, right).

In the abducens nucleus, vGlut2-immunopositive puncta were found along the somatic membrane of all three types of neurons (Fig. 4b, c). However, MIF motoneurons had significantly fewer somatic glutamatergic inputs than SIF motoneurons (*p* < 0.001, *t*-test) per membrane length (Figs. 4b, red arrowheads, 6a, left). The density of vGlut2-immunopositive puncta per membrane length of INTs was similar to and not significantly different (*p* = 0.43, *t*-test) from, that of SIF motoneurons (Figs. 4c, blue arrows, 6a, left; Table 3).

**Table 3:** Qualitative summary of immunohistochemical results (**−** no labeling, + weak, +  + moderate, +  +  + strong labeling)

	Abducens			Trochlearis	
	MIF	SIF	INT	MIF	SIF
Kv1.1	+	+ + +	+ + +	+	+ + +
Kv3.1b	−	+ +	+ + +	−	+ +
Nav1.6	+	+	+	+	+
KCC2	+	+	+	+	+
Cav3.1	+ + +	−	−	+ + +	+/−
Cav3.2	+	+ +	+ +	+	+ +
Cav3.3	+	+	+	+	+
vGlut1	−	−	−	−	−
vGlut2	+ +	+ + +	+ + +	+ +	+ + +
GlyT2	+	+ +	+ +	−	−
GAD 65/67	+	+ +	+ +	+	+
GluR2/3	+	+ +	+ +	+	+ +
NMDAR1	−	+	+ + +	−	+
GlyR1a	+	+	+	−	−

The number of vGlut2-immunopositive puncta on both MIF and SIF motoneurons appeared to be similar between the trochlear and abducens nucleus (Figs. 4b, 5c, red arrowheads and green arrows). In fact, quantification of the magnitudes did not reveal any significant difference in the extent of the vGlut2-immunopositivity around the somata of motoneurons in the trochlear nucleus, as compared to those encountered in the abducens nucleus (Fig. 6a, left). This was true for MIF (*p* = 0.11, *t*-test) and for SIF motoneurons (*p* = 0.12, *t*-test).

#### Differential expression of glutamatergic receptors by MIF and SIF motoneurons in the abducens and trochlear nuclei

Ionotropic AMPA receptors, composed of several different subunits, cause a Na^+^ influx (and K^+^ efflux) upon glutamate binding, thereby exciting the neuron. The additional permeability for Ca^2+^ ions is prevented by the presence of GluR2 subunits, as a component of the AMPA receptor (Wollmuth 2018). Therefore, antibodies directed against the subunits GluR2 and GluR3 (GluR2/3) were used to test the presence of these calcium-impermeable AMPA receptors in abducens and trochlear neurons. GluR2/3-immunolabeling was encountered in the neuropil and along the membrane of neurons in both motor nuclei (Figs. 4d, 5d). While immunolabeling was more intense around SIF motoneurons and INTs within the core region of the abducens nucleus (Fig. 4e, green and blue arrows, respectively; Table 3), MIF motoneurons located at the medial border expressed a weaker labeling (Fig. 4e, red arrowheads). This differential labeling pattern was even more pronounced for SIF and MIF motoneurons in the trochlear nucleus (Fig. 5e, green arrows and red arrowheads, respectively), where the intensity of the GluR2/3-immunolabeling clearly subsided towards the dorsal cap, which contains MIF motoneurons.

Ionotropic NMDA receptors allow the influx of Ca^2+^-ions in addition to Na^+^ and K^+^ exchange across the membrane upon glutamate binding and thus promote more extended postsynaptic responses than AMPA receptors (Dingledine et al. 1999). Therefore, immunolabeling of the NMDAR1 subunit was evaluated in neurons of the abducens and trochlear nuclei (Figs. 4f, g, 5f, g; Table 3). In the abducens nucleus, the strongest NMDAR1-immunostaining was found along the somatic and dendritic membrane of ChAT-immunonegative INTs (Fig. 4f, g, blue arrows). Albeit much less abundant as compared to INTs, NMDAR1 was also present along the membrane of SIF motoneurons in punctate form (Fig. 4g, green arrow), suggesting the presence of numerous synapses endowed with this glutamate receptor subtype. In contrast, MIF motoneurons failed to show any NMDAR1-immunolabeling (Fig. 4g, red arrowhead). NMDAR1-immunolabeling in the trochlear nucleus was similar to that of the abducens nucleus with clear punctate immunolabeling associated with SIF motoneuronal membranes (Fig. 5f, g, green arrows), whereas MIF motoneurons were clearly spared by NMDAR1-immunolabeling (Fig. 5f, g, red arrowheads).

### Inhibitory transmitters and receptors in neurons of the abducens and trochlear nuclei

#### Differential glycinergic and GABAergic inputs to abducens and trochlear neurons

**Fig. 7:**
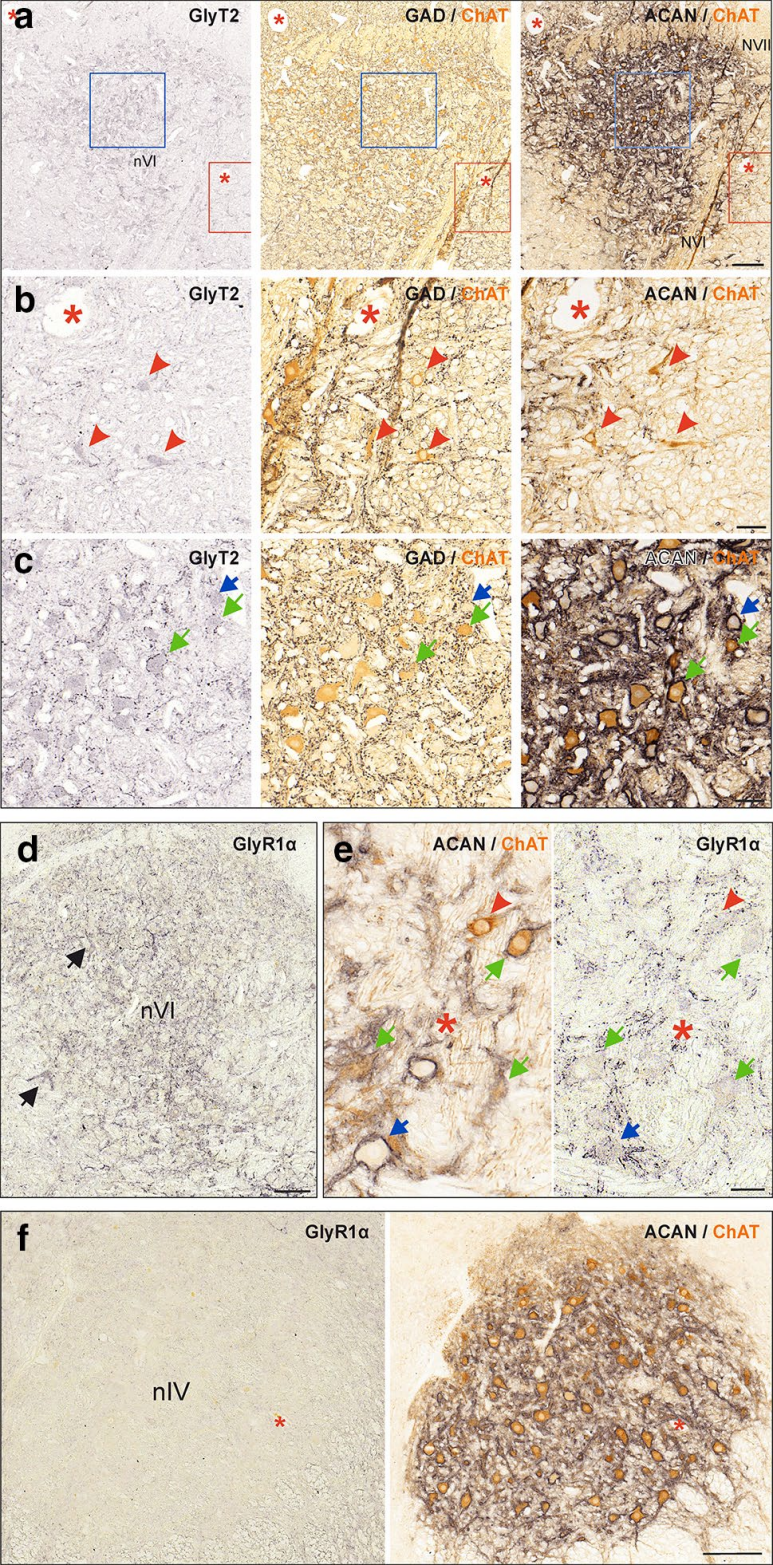
Inhibitory transmitter profile of synapses onto abducens and trochlear neurons. **a**–**c** Consecutive coronal paraffin sections illustrating presynaptic glycinergic (left) and GABAergic terminals (middle; black) onto choline acetyltransferase (ChAT)- and aggrecan (ACAN)-immunopositive (right) SIF MNs (**c**, green arrows), onto ACAN-immunonegative MIF MNs (**b**, red arrowheads) and onto ChAT-immunonegative INTs (**c**, blue arrow) in the abducens nucleus (nVI). Colored boxes indicate the areas illustrated at higher magnification in b (red) and c (blue), respectively. Immunolabeling for the glycine transporter 2 (GlyT2) in an overview (**a**) and higher magnification (**b**,**c**, left, black) reveals few glycinergic synapses on MIF MNs (**b**, red arrowheads), and more numerous synapses on SIF MNs (**c**, green arrows) and INTs (**c,** blue arrows) in nVI. Immunolabeling for GAD in an overview (**a**, middle) and higher magnification (**b**,**c**, middle) in nVI shows fewer GAD-immunopositive terminals on MIF MNs (**b**, red arrowheads), compared to more abundant terminals on SIF MNs (**c**, green arrows) and INTs (**c**, blue arrow). **d**–**f** glycine receptor (GlyR1α)-immunolabeling in the nVI and the trochlear nucleus (nIV). Only nVI neurons express GlyR1α that is visible as punctate staining (**d, e**, right). Note the stronger dendritic than somatic immunolabeling (**d**, black arrows) more clearly seen in the close-up (**e**) with GlyR1α-immunopositive puncta along the membrane of SIF (green arrows), MIF MNs (red arrowheads) and INTs (blue arrows). Note that GlyR1α-immunolabeling is absent in nIV neurons (**f**). Scale bar represents 200 μm in **a, d, f,** and 50 μm in **b, c, e**

A previous study reported a comparable extent of GABA-ergic inputs to trochlear MIF and SIF motoneurons, while glycinergic inputs to both motoneuronal types were absent (Zeeh et al. 2015). Consequently, the different abducens neuron types were analyzed here for the presence of the respective inhibitory synaptic structures. Glycinergic and GABAergic inputs to abducens neurons were visualized on consecutive 5 μm sections through immunolabeling of the glycine transporter 2 (GlyT2) and glutamate decarboxylase (GAD), respectively (Fig. 7; Table 3). Numerous GlyT2- and GAD-immunopositive puncta were distributed throughout the abducens nucleus (Fig. 7a–c, left and middle columns, respectively). The weak background labeling of the somata found by GlyT2-immunostaining was used for proper identification and visualization of the same neurons on adjacent sections (Fig. 7a–c, left column).

Using cell types determined by ChAT and ACAN staining (Fig. 7a–c, right), we found that while GlyT2-immunopositive puncta were present to a comparable extent along the somatic membrane of SIF motoneurons and INTs in the abducens nucleus (Fig. 7c, left column; green arrows and blue arrows, respectively), considerably fewer puncta were observed along the somatic membrane of MIF motoneurons (Fig. 7b, left column, red arrowheads). Quantification revealed a two to three-fold (240.7%; *p* < 0.001, *t*-test) difference between average numbers of GlyT2-immunopositive puncta surrounding SIF motoneurons as compared to MIF motoneurons (Fig. 6c). INTs had a significantly higher number (10.2%; *p* < 0.05, *t*-test) of glycinergic inputs as compared to SIF motoneurons (Fig. 6c). In general, GlyT2-immunopositive puncta were observed in greater abundance on the dendrites of MIF motoneurons, however, quantitative confirmation of differences between somatic versus dendritic labeling was not possible using 5 μm paraffin sections.

The pattern of GAD-immunolabeling in the abducens nucleus was similar to that of GlyT2-immunolabeling. GAD-immunopositive puncta were abundant around the somata of SIF motoneurons and INTs, while fewer puncta were found around MIF motoneurons (Fig. 7b, c, middle column, green arrows, blue arrow and red arrowheads, respectively). Quantification revealed up to three times (279.1%; *p* < 0.01, *t*-test) as many GAD-immunopositive puncta surrounding SIF motoneurons as compared to MIF motoneurons (Fig. 6b). With respect to GlyT2-immunopositive puncta, INTs were consistently surrounded by a significantly higher number of GAD-immunopositive puncta (18.7%; *p* < 0.01, *t*-test) as compared to SIF motoneurons (Fig. 6b).

To compare the extent of GAD-immunostaining around abducens and trochlear neurons, immunohistochemistry was performed on trochlear motoneurons using consecutive 5 μm paraffin sections of the same case (M5). The respective analysis yielded GAD-immunopositive puncta on both MIF and SIF motoneurons (not shown) in agreement with a previous report (Zeeh et al. 2015). Quantitative analyses of MIF and SIF trochlear motoneurons demonstrated significantly fewer (*p* < 0.01, *t*-test) GAD-immunopositive puncta around MIF as compared to SIF motoneurons (Fig. 6b).

#### Differential expression of glycine receptor 1α in abducens and trochlear neurons

The differential organization of glycinergic synaptic structures innervating MIF and SIF abducens motoneurons were complemented by an evaluation of the expression of the glycine receptor subunit 1α. Immunostaining with antibodies against GlyR1α (clone mAb4a, see Methods; Table 2) yielded punctate labeling throughout the abducens nucleus (Fig. 7d), while such staining was entirely absent in the trochlear nucleus in agreement with a lack of glycinergic inputs to trochlear motoneurons (Fig. 7f; Table 3). In the abducens nucleus, all neuronal subtypes, expressed a stronger dendritic than somatic immunolabeling (Fig. 7d, black arrows; Fig. 7e, right). More-over, numerous immunoreactive puncta were observed in the neuropil of the nucleus. Importantly, both MIF and SIF motoneurons revealed a somatic punctate GlyR1α-labeling, however, with varying abundance within the respective populations (Fig. 7e, red arrowheads and green arrows, respectively).

#### Potassium-chloride co-transporter (KCC2) has a similar expression pattern in the abducens and trochlear nuclei

**Fig. 8:**
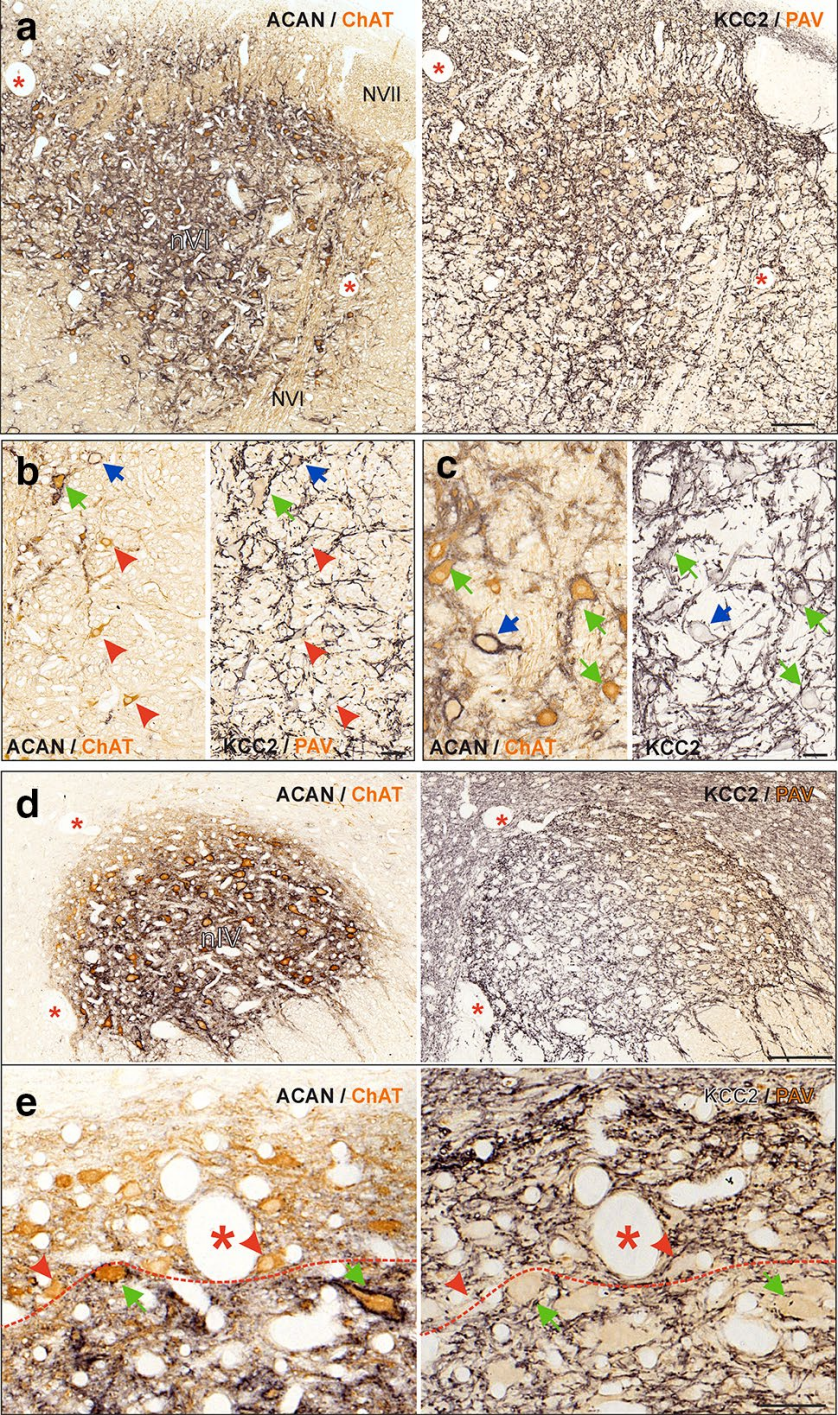
Chloride-potassium co-transporter (KCC2) in abducens and trochlear neurons. **a**–**e** Consecutive paraffin sections through the abducens (nVI) (**a**–**c**) and trochlear nucleus (nIV) (**d, e**) immunostained for KCC2 (right) and combined aggrecan (ACAN)-based perineuronal nets (PN; black) and choline acetyltransferase (ChAT; brown, right). **b, c** Close-up of KCC2 (black) expression in MIF (red arrowheads) and SIF motoneurons (MNs) (green arrows) (**b**) and of INTs (blue arrow) (**c**). **e** Close-up of KCC2-immunolabeling in MIF MNs (red arrowheads) within the dorsal cap of nIV and SIF MNs (green arrows) within the core region of nIV. Note the intense labeling along the dorsal border of the trochlear nucleus in (**d**,**e**). Red dashed lines indicate the tentative position of the border delineating the dorsal cap of nIV. The details in **b, c, e** were obtained from different sections as those shown in **a,d**. Scale bar represents 200 μm in **a, d**, and 50 μm in **b, c,e**

The characterization of the inhibitory transmitter profile of MIF and SIF motoneurons was complemented by classifying the presence of the potassium-chloride symporter 2 subunit (KCC2). Accordingly, immunoperoxidase staining against KCC2 was combined with immunostaining for either ChAT or parvalbumin (PAV) as motoneuronal markers in both motor nuclei (Fig. 8). Because KCC2 co-localizes with both glycinergic and GABAergic receptors (Chamma et al. 2012), a potentially differential expression in abducens and trochlear neurons could provide further insight into the regulatory mechanisms provided by different types of inhibitory inputs. The immunostaining revealed that all three types of abducens neurons were labeled by the KCC2 antibody (Fig. 8a–c; Table 3). While the immunolabeling of the somatic membrane was weak, the labeling became gradually more intense towards the distal dendrites (Fig. 8b, c, right columns), strongly coinciding with the labeling pattern of the GlyR1α antibody (Fig. 7d, e, right column). In contrast, KCC2-immunolabeling was absent along the axons that form the abducens nerve root (Fig. 8a, NVI), in agreement with the reported properties of this protein (Chamma et al. 2012). In the trochlear nucleus, MIF and SIF motoneurons exhibited a KCC2-immunoreactivity that was comparable in extent with the similarly graded soma-dendritic increase in the intensity of abducens neurons (Fig. 8d, e). The labeling was much more intense at the outermost border of the trochlear nucleus, outside the dorsal cap that contains MIF motoneurons (Fig. 8d, e).

### MIF and SIF motoneurons differ in low voltage-activated calcium channel profiles

**Fig. 9:**
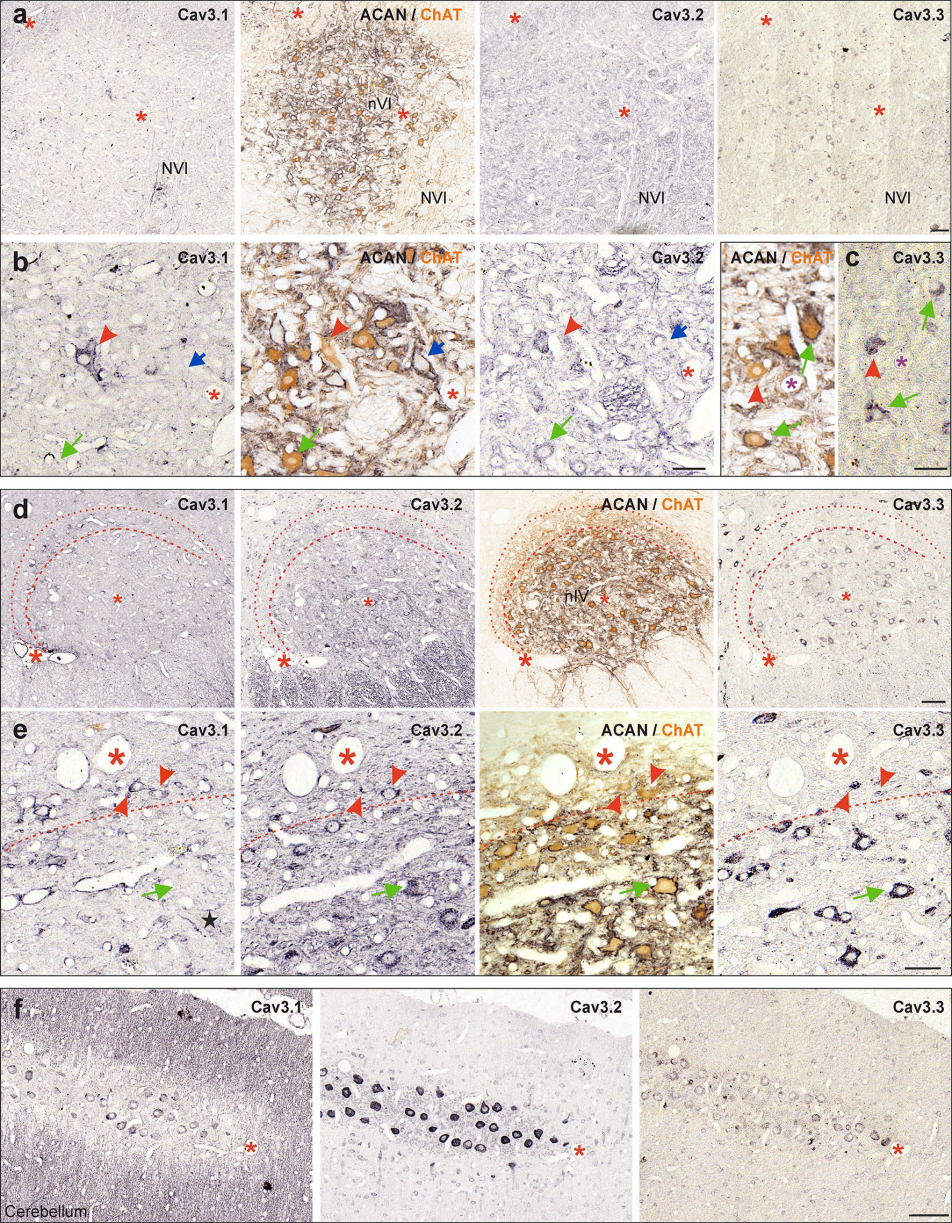
Low-voltage activated calcium channel family (Cav3) subunits in the abducens and trochlear nucleus. **a** Consecutive coronal paraffin sections through the abducens nucleus (nVI) (**a**) depicting the immunolabeling for Cav3.1 (first panel), Cav3.2 (third panel) and Cav3.3 (fourth panel) subunits with combined immunostaining for ChAT (brown) and ACAN (black) (second panel) as reference. **b, c** Close-up of Cav subunit expression in nVI MIF (red arrowheads) and SIF motoneurons (MNs) (green arrows) and INTs (blue arrow). **d** Consecutive coronal paraffin sections through the trochlear nucleus (nVI) depicting the immunolabeling for Cav3.1 (first panel), Cav3.2 (second panel) and Cav3.3 (fourth panel) subunits with combined immunostaining for ChAT (brown) and ACAN (black) (third panel) as reference. Thin dashed lines in **d** indicate the border of nIV and thick dashed lines indicate the boundary between the MIF and SIF MNs. **e** Close-up of Cav subunit expression in MIF (red arrowheads) and SIF MNs (green arrow) on different sections as those illustrated in **d**. Note the weak Cav3.1 expression along the membrane of some SIF MNs (left column, black star). Red dashed lines indicate the tentative position of the border delineating the dorsal cap of nIV. **f** Cerebellar Purkinje cells located on the same consecutive sections as nVI as controls for immunopositivity of the different Cav subunits. Scale bar indicates 100 μm in **a, d, f** and 50 μm in **b, c, e**

The expression of members of the voltage-gated calcium channel-3 family (low-voltage activated channels), which are generally involved in neuronal excitability, pace-making and repetitive firing (Zamponi et al. 2015) (Fig. 9; Table 3), were investigated with respective antibodies against all three members of the Cav3 family (Cav3.1–3.3). The expression pattern was assessed in the three sections adjacent to a section treated with antibodies against ChAT and PN, which again served as a reference for MIF and SIF motoneurons. At low magnification, we observed sparse immunolabeling for Cav3.1 subunit (Fig. 9a, first column), whereas Cav3.2 yielded weak somatic immunoreactivity (Fig. 9a, third column) in the abducens nucleus. We utilized Cav3.1- and Cav3.2-immunostaining in Purkinje cells as a direct internal control for the degree of positive staining (Fig. 9f). In contrast, Cav3.3-immunolabeling in abducens neurons was qualitatively comparable to that of the Purkinje cells (Fig. 9a, c, f, last columns).

Closer inspection of the labeling pattern of the three family members revealed that the somatic membrane of MIF abducens motoneurons contained the Cav3.1 subunit (Fig. 9b, red arrowheads), whereas no labeling was found in SIF motoneurons and INTs (Fig. 9b, left column, green and blue arrows, respectively). In contrast, somatic Cav3.2, as well as Cav3.3-immunolabeling, was encountered in all three abducens neuronal subtypes (Fig. 9b, c, INTs are not illustrated in Fig. 9c). The assessment of Cav3 channels in the trochlear nucleus yielded qualitatively similar results (Fig. 9d, e). Accordingly, intense Cav3.1-immunolabeling was encountered in trochlear MIF motoneurons, located within the dorsal cap of the nucleus (Fig. 9d, between red dashed lines, e, first columns, red arrowheads), while only a few SIF motoneurons within the core area of the nucleus expressed weak labeling along the somatic membrane (Fig. 9e, first column, green arrow, black star). In contrast, Cav3.2- and Cav3.3-immunolabeling were detected in both MIF and SIF trochlear motoneurons (Fig. 9d, e, second and last columns, red arrowheads and green arrows, respectively). Collectively, these results suggest a differential contribution of the three subtypes of voltage-activated calcium channels to the excitation of neurons in both the abducens and trochlear nucleus.

## Discussion

**Fig. 10:**
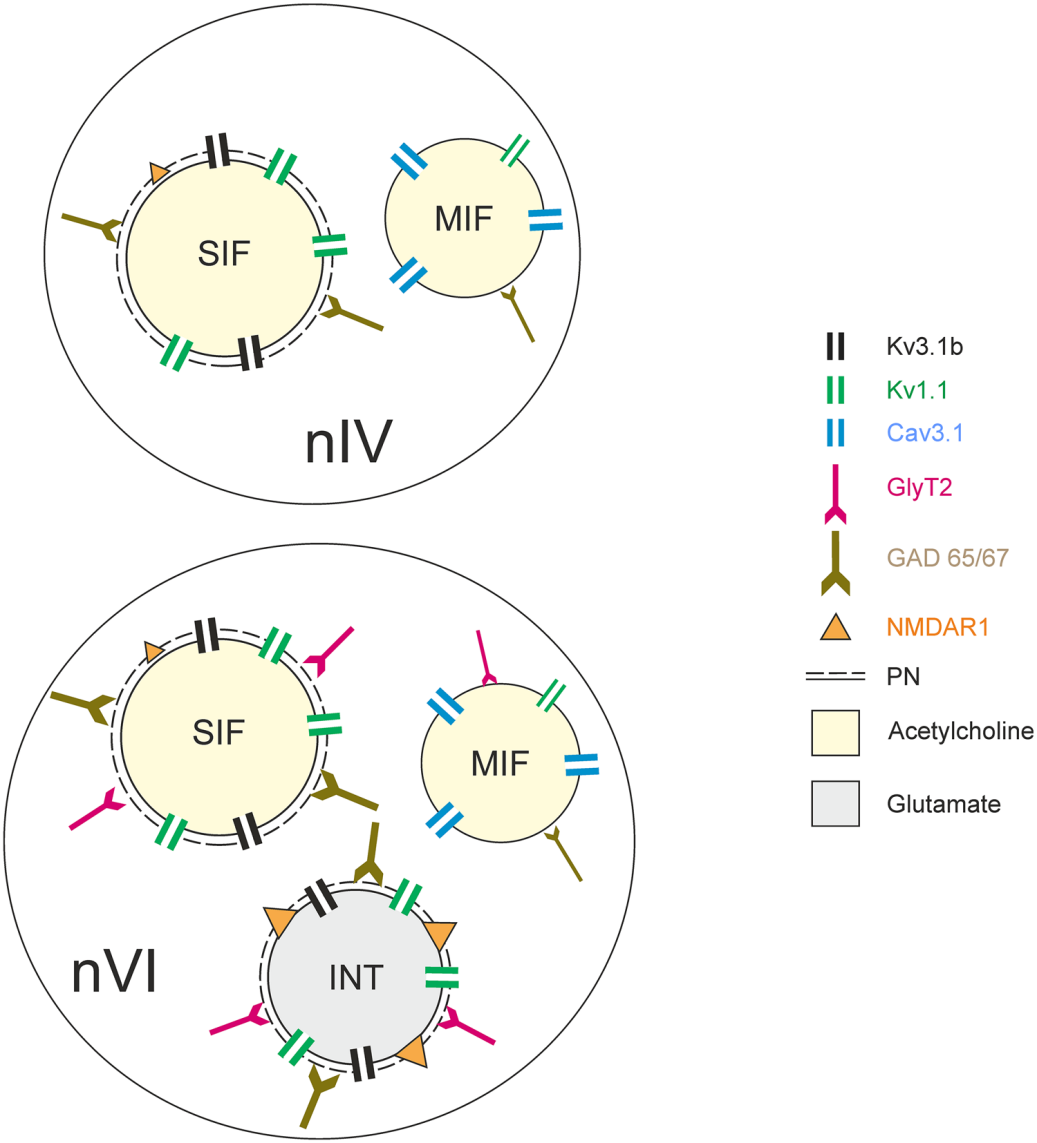
Summary diagram depicting differential molecular signatures of trochlear and abducens neurons. Schematic representation delineating predominating ion channels, transmitters and transmitter receptors of the different sets of trochlear (upper) and abducens neurons (lower). The relative strength of the immunostaining is indicated by the size and number of the respective symbols

This study explored the basis for the functional properties of different groups of neurons in the abducens and trochlear nuclei in macaque monkey using immunohistochemistry with an emphasis on motoneurons of MIF and SIF muscle fibers, as shown in the summary figure (Fig. 10). Differences between MIF and SIF motoneurons with respect to voltage-gated potassium channel subunits Kv1.1 and Kv3.1b and low-voltage activated calcium channel subunit Cav3.1 were particularly prominent. This pattern suggests that MIF motoneuron membrane properties are preferentially suitable for the generation of low-dynamic motor commands. Moreover, different extents of synaptic inputs to cell bodies of MIF in comparison to SIF motoneurons were discovered for both excitatory and inhibitory inputs. This is in line with smaller numbers of synaptic contacts on somata and proximal dendrites of MIF-versus SIF-motoneurons of the medial rectus muscle (Erichsen et al. 2014). Together with differences in the glutamatergic receptor profile of MIF and SIF motoneurons (GluR2/3 and NMDAR1), the present findings suggest that the motor control of extraocular muscles is achieved through a combination of distinct intrinsic motoneuronal membrane properties and differentially organized synaptic inputs. Comparative observations for abducens internuclear neurons revealed that they display stronger immunolabeling for Kv3.1b, perineuronal nets and NMDAR1 as compared to SIF motoneurons, along with higher inhibitory synapse densities (see Table 3 and summary diagram in Fig. 10). Collectively, these findings may account for the previously observed enhanced excitability and earlier recruitment of abducens internuclear neurons.

### Differences in connectivity and intrinsic membrane properties of MIF and SIF motoneurons

#### Different transmitter profiles of premotor inputs to MIF and SIF motoneurons in the abducens and trochlear nuclei

Glycinergic inputs to the abducens nucleus generally derive from multiple sources including inhibitory second-order vestibulo-ocular neurons in the ipsilateral medial vestibular nucleus (MVN), inhibitory burst neurons (IBNs) of the horizontal saccadic circuitry and possibly neurons in the contralateral nucleus prepositus hypoglossi (PPH) (Horn 2006; Spencer et al. 1989; Straka and Dieringer 1993). However, based on retrograde transsynaptic tracing studies, identified premotor neurons of abducens motoneurons, such as saccadic IBNs and second-order vestibulo-ocular neurons constitute a major source for glycinergic inputs to SIF, but not to MIF, motoneurons (Ugolini et al. 2006). Therefore, lower quantities of GlyT2-immunopositive puncta localized on MIF as compared to SIF abducens motoneurons can be explained by fewer glycinergic inputs from IBNs. However, the current study cannot rule out a differential organization of respective inputs to MIF and SIF abducens motoneurons from other sources. In the trochlear nucleus, the lack of GlyT2-positive puncta (not shown) confirms previous findings (Zeeh et al. 2015). This was expected, as inhibition from premotor neurons during saccades, VOR and gaze-holding is mediated by different transmitters for horizontal and vertical eye movements, not only in primates but also in other vertebrates including amphibians (Horn and Straka 2021; Soupiadou et al. 2018). According to this scheme, glycine is used in pathways for horizontal eye movements, and GABA is used in circuits comprising the vertical/oblique ocular motor system. This may be due to a differential anatomical origin of the presynaptic neurons and a differential distribution of these two transmitters along the longitudinal axis of the brainstem (Spencer and Baker 1992; Spencer et al. 1989; Straka et al. 2014).

GABAergic inputs to the abducens nucleus may originate from internuclear neurons in the oculomotor nucleus as demonstrated in cat (De la Cruz et al. 1992; Maciewicz et al. 1975). Stimulation and recording experiments in primates mainly revealed a crossed excitatory projection of oculomotor internuclear neurons to the abducens nucleus, consistent with a role in horizontal conjugate eye movements (Clendaniel and Mays 1994). The finding of a few ipsilaterally projecting oculomotor internuclear neurons that decrease their discharge during adduction of the ipsilateral eye indicates a likely auxiliary inhibitory projection to abducens neurons (Clendaniel and Mays 1994).

GABAergic afferents to the trochlear nucleus originate from second-order vestibulo-ocular neurons in the ipsilateral superior vestibular nucleus (SVN), which provide an inhibition following anterior semicircular canal stimulation (McElligott and Spencer 2000; Precht et al. 1973; Soupiadou et al. 2018) and primarily target SIF motoneurons (McCrea et al. 1987; Ugolini et al. 2006). Additional GABAergic inputs may arise from the ipsilateral dorsal Y-group, and provide an inhibition to superior oblique and inferior rectus motoneurons during upward smooth pursuit eye movements (Chubb and Fuchs 1982; Zeeh et al. 2020). GABAergic projections to the trochlear nucleus may also include inhibitory burst neurons for vertical saccades, located in the ipsilateral interstitial nucleus of Cajal (monkey: Horn et al. 2003) and in the rostral interstitial nucleus of the medial longitudinal fasciculus (RIMLF) (cat: Spencer and Wang 1996). In contrast to a previous report (Zeeh et al. 2015), a differential density of GAD-immunopositive terminals was encountered in the present study for MIF and SIF motoneurons (Fig. 6b). This discrepancy could simply be due to the use of thicker paraffin sections in the former study, which might have reduced the chance of evaluating overlapping puncta and/or reduced penetration of the antibodies. Nevertheless, the difference in GAD-immunopositive terminal density between SIF and MIF motoneurons was generally much lower for trochlear than for abducens motoneurons.

Glutamatergic inputs to abducens neurons, demonstrated by vGlut2-immunoreactive puncta, may arise from excitatory VOR neurons in the contralateral MVN, or ventral lateral vestibular nucleus (Delgado-García et al. 1986; McElligott and Spencer 2000), from excitatory premotor burst neurons located in the ipsilateral PPRF (Horn 2006; Ugolini et al. 2006) and from non-cholinergic INTs located primarily in the contralateral oculomotor nucleus (Clendaniel and Mays 1994). As revealed by retrograde transsynaptic tracer studies, burst neurons in the PPRF and magnocellular neurons in the MVN project predominantly to SIF motoneurons (Ugolini et al. 2006; Wasicky et al. 2004). The results of the current study demonstrated the presence of about 50% more vGlut2-positive puncta per length of somatic membrane for SIF motoneurons compared to MIF motoneurons in the abducens nucleus (Fig. 6a, left). Whether or not additional projections from the PPRF are responsible for this difference was impossible to determine with the employed method. Moreover, it is also difficult to predict the functional consequences of the different extents of glutamatergic synapses for the overall excitability, and thus the excitability of MIF and SIF motoneurons, given the obvious morphological differences (Torres-Torrelo et al. 2012). The more intense immunolabeling of vGlut2 on motoneuronal dendrites as compared to the somatic membrane potentially has functional implications in terms of spatiotemporal integration of excitatory signals, assuming an electrotonic compartmentalization (Magee 2001; Rekling et al. 2000).

The trochlear nucleus receives monosynaptic inputs from excitatory premotor burst neurons in the ipsilateral RIMLF mediating downward saccades (Horn and Büttner-Ennever 1998; Moschovakis et al. 1991; Spencer and Wang 1996). In the cat, these projections use glutamate and/or aspartate as transmitter (Spencer and Wang 1996), and may therefore contribute to the vGlut2-immunopositive terminals observed on SIF motoneurons. In addition, part of the latter excitatory synapses likely derives from glutamatergic projections of second-order vestibular neurons located in the contralateral MVN and in the interstitial nucleus of Cajal, as suggested previously (Zeeh et al. 2015). Finally, weak ipsilateral excitatory projections to trochlear motoneurons originate in the PPH (Baker et al. 1977). Interestingly, vGlut2-immunopositive synaptic structures around the motoneurons were more intense in the trochlear than in the abducens nucleus, and the comparison between MIF and SIF motoneurons in the trochlear nucleus yielded smaller differences in immunopositive puncta per length of the somatic membrane (Fig. 6a, left). The more pronounced differentiation between MIF and SIF abducens motoneurons with respect to vGlut2-immunopositive synaptic structures might be related to the larger spectrum and dynamic diversity of horizontal (i.e., vergence, saccades) as compared to torsional eye movements encoded, at least in part in the trochlear nucleus.

#### Impact of transmitters on the neuronal excitability of MIF and SIF motoneurons

Glutamate, as the major excitatory neurotransmitter in the central nervous system has been shown to also depolarize oculomotor motoneurons (Durand et al. 1987; Torres-Torrelo et al. 2012). While the activation of glutamatergic AMPA receptors generates a fast-rising response, a relatively slower dynamic of the depolarization is achieved by NMDA receptors, as demonstrated for cat abducens motoneurons (Durand et al. 1987). Glutamate modulates the firing rate of extraocular motoneurons by decreasing the voltage threshold depending on cell size and eye position-related recruitment thresholds (Torres-Torrelo et al. 2012). Importantly, glutamate has a larger impact on the phasic as compared to the tonic component of the motoneuronal discharge (Torres-Torrelo et al. 2012) and thereby is capable of regulating the firing pattern and rate of motoneurons, which transmit strong phasic components. However, the phasic component/burst additionally requires intrinsic active membrane properties as a decisive factor for fast firing rate alterations, as demonstrated for spinal motoneurons (Iwagaki and Miles 2011; Torres-Torrelo et al. 2012). Therefore, the glutamatergic control of motoneuron discharge is probably achieved by a combination of respective transmitter receptor profiles and differential sodium/potassium channel expression patterns.

Glutamatergic synaptic transmission in the abducens and trochlear nuclei involves vGlut2, but not vGlut1, in agreement with the complementary expression of both transporter subtypes and predominant presence of vGlut2 in the brainstem (Fremeau et al. 2001, 2004). Although vGlut2 is associated with a higher transport probability and faster synaptic release in highly active circuits, as compared to vGlut1, vGlut2-mediated transmission does not necessarily imply higher firing rates in the target neuron (Fremeau et al. 2004). The lack of any terminals expressing vGlut1 in the abducens and trochlear nuclei confirms and extends previous observations that only the subpopulation of medial rectus MIF motoneurons in the oculomotor nucleus is targeted by vGlut1-positive terminals (Zeeh et al. 2015). The cell specificity of glutamatergic transmission may be due to the lower dynamic requirements of MIF motoneurons during convergence eye movements. In contrast, SIF motoneurons express significantly more vGlut2-immunopositive puncta per circumferential somatic membrane than MIF motoneurons, and this transporter subtype occurs at an even higher density on dendritic membranes. The recruitment of motoneurons depends on the input resistance, which as a rule of thumb is inversely correlated with the neuronal surface area. This suggests that similar amounts of excitatory synaptic elements depolarize the smaller MIF motoneurons more efficiently (Henneman et al. 1965; Magee 2001; Mendell 2005; Torres-Torrelo et al. 2012). However, fewer glutamatergic synapses on MIF motoneurons might cause less efficient membrane potential alterations given the smaller size of the glutamatergic synaptic structures (Fig. 6a). Alternatively, differential expression patterns of voltage-gated potassium channels could produce different neuronal outputs from MIF and SIF motoneurons, collectively rendering a clear prediction of the findings on net firing rate changes at best difficult (see Perineuronal nets, potassium channels and fast-spiking section).

### Control of neuronal excitability by intrinsic membrane properties of MIF and SIF motoneurons

#### Transmitter receptors and their functional impact on selective vulnerability in neurodegenerative diseases

Ionotropic glutamate receptors consist of AMPA/kainate and NMDA receptors. Increasing evidence indicates that NMDA receptors mediate slower and more prolonged responses, whereas AMPA receptors initiate more rapid and transient responses (Traynelis, 2010). Unlike AMPA receptors, which increase the permeability mainly for sodium and potassium, NMDA receptors are also permeable for Ca^2+^, producing prolonged depolarizations (Dingledine et al. 1999). In the abducens nucleus of larval *Xenopus laevis*, motoneurons were subdivided into two functional subgroups with respect to the pharmacological profile of vestibular excitatory inputs (Dietrich et al. 2017). One population of motoneurons is mainly activated through AMPA receptors, while motoneurons of the second subgroup receive vestibular excitatory inputs predominantly through NMDA receptors (Dietrich et al. 2017). The present study revealed lower expression levels of GluR2/3- and a lack of NMDAR1-immunoreactivity in MIF motoneurons, whereas immunostaining for both receptors was present in SIF motoneurons. However, no indication for a potential distinction into subgroups with respect to ionotropic glutamate receptor expression was encountered in either of the two motor nuclei examined. This does, however, not exclude the possibility that histochemical analyses of other glutamate receptor subunits, including a more detailed quantification, would allow a dissociation into particular subcategories of SIF and MIF motoneurons.

Differential glutamatergic receptor expression in MIF and SIF motoneurons has important functional and clinical implications. First, fewer vGlut2-immunopositive terminals with fewer GluR2/3 and no NMDAR1 expression by MIF motoneurons provides suggestive evidence for lower synaptic sensitivity in response to glutamate, as compared to SIF motoneurons. Second, this could render MIF and SIF motoneurons differentially resistant to glutamate-induced excitotoxicity as found, for instance, in amyotrophic lateral sclerosis (ALS) (Brockington et al. 2013). The higher expression levels of AMPA-receptors containing the GluR2 subunit, which renders extraocular motoneurons less permeable to calcium, potentially represents one cause for the relative resistance of the ocular motor system to degeneration in ALS as compared to spinal motoneurons (Van Damme et al. 2005). However recent studies imply that the ocular motor system is not completely spared in ALS, but it may affect motoneurons differentially. Therefore, the comparably lower GluR2/3 expression in MIF motoneurons along with the absence of calcium-binding proteins (Eberhorn et al. 2005) could constitute one factor for the putatively higher vulnerability of MIF motoneurons as compared to SIF motoneurons, consistent with the observed degeneration of MIFs in ALS patients (Ahmadi et al. 2010; Tjust et al. 2017).

Control of motoneuronal excitability is also maintained by inhibitory transmitters through fast-acting ionotropic glycinergic and GABA_A_ receptors, which are permeable for Cl^−^ upon ligand binding (Rekling et al. 2000). Glycine and GABA_A_ receptors decrease excitability, depending on the intracellular Cl^−^ concentration, which is regulated in part by the activity of member 5 of KCC2 (Chamma et al. 2012). KCC2-immunolabeling of MIF and SIF motoneurons, therefore, complies with requirements for glycinergic and GABAergic neurotransmission. Similar motoneuron membrane localizations of GlyR1 and KCC2 suggest that they co-localize, with weak somatic labeling and stronger labeling of the dendrites (Figs. 7d, 8a–c). In addition, GABA_A_ receptors have been demonstrated to also colocalize both with GlyR1 (Todd et al. 1996) and KCC2 (Huang et al. 2013). KCC2 determines the polarity and efficacy of GABA_A_ and glycine receptors, and pathologies that derive from a KCC2 deficiency cause neural excitability-related disorders, such as epilepsy and Huntington’s disease (Tang 2020).

#### Perineuronal nets, potassium channels and fast-spiking

Beyond a potential role in neuroprotection and regulating plasticity, PNs are associated with parvalbumin-immunopositive fast-spiking interneurons, where this structural element presumably acts as cation buffer by providing a negatively charged trap for sodium and potassium ions around the cell membrane (Härtig et al. 1999). Moreover, PNs are correlated with the expression of the voltage-gated potassium channel Kv3.1b, potentially enhancing firing characteristics of fast-spiking neurons via regulation of Kv3.1b and Kv1.1 expression and recruitment (Favuzzi, 2017; Härtig et al. 1999). Low voltage-activated members of the Kv1 family are known to regulate the resting membrane potential, spike threshold and neuronal excitability (Johnston et al. 2010). A recent study in mouse deep cerebellar nucleus neurons demonstrated that application of specific Kv1 channel blockers lowers action potential threshold, increases spontaneous firing rate (or introduces spontaneous tonic firing in previously silent neurons) and reduces the fast-spiking capacity (Feria Pliego and Pedroarena 2020). On the other hand, members of high-voltage activated Kv3 channels open during action potentials, thereby shortening spike duration by interfering with the repolarization (Johnston et al. 2010). Together, Kv1.1 and Kv3.1b enhance sustained and high-frequency firing by minimizing Nav inactivation (Gu et al. 2018; Kaczmarek and Zhang 2017; Kodama et al. 2020).

Recent findings in cat indicated that both MIF and SIF motoneurons express phasic-tonic discharge patterns and contribute to all types of eye movements (Hernández et al. 2019). This appears to conflict with the fact that MIF motoneurons lack the Kv3.1 ion channel subunit, which is a reliable marker of fast-spiking neurons capable of bursts (Kodama et al. 2020), along with lower levels of Kv1.1-immunoreactivity. Additionally, the graded immunolabeling of Kv1.1 of abducens and trochlear neurons is interesting due to the role in regulating spike threshold, and therefore overall excitability. However, varying immunolabeling intensities cannot be translated directly into differential mRNA content or protein expression. Nonetheless, varying Kv1.1 expression levels comply with the observation that extraocular motoneurons appear to form a functionally continuous spectrum with regard to eye position and eye velocity-related recruitment thresholds (Hernández et al. 2019; Nieto-Gonzales et al. 2007). Therefore, Kv1.1 could be a contributor to the electrophysiological substrate required for regulating neuronal firing threshold and corresponding eye movement-related sensitivity. This suggestion places MIF and SIF motoneurons on opposite ends of a population spectrum with distinct histochemical profiles that correspond to neurons with relatively tonic or relatively phasic firing characteristics, respectively (Davis-López de Carrizosa et al. 2011; Horn and Straka 2021).

#### Fast-firing and associated ion channel expression profile

In a recent study conducted on MVN neurons with different firing characteristics in mice, Kodama et al. (2020) identified distinct co-expression patterns of genes, including (but not limited to) voltage-gated ion channel families, neurofilaments and calcium-binding proteins. An important discovery was the strong correlation of ion channel expression including Kv1.1, Kv3.1 and Nav1.6 in the fastest-spiking MVN neurons that constitute a major synaptic input to extraocular motoneurons (Kodama et al. 2020). These members of the ‘fast spiking gene module’ are potentially co-regulated and responsible for the persistence of a high excitability during repetitive firing (Kodama et al. 2020). Persistent high firing rates, such as found in ocular motor circuitries, require minimal Nav inactivation, which is facilitated by Kv1.1 and Kv3.1 subunits (Carter and Bean 2011; Foust et al. 2011; Kaczmarek and Zhang 2017). Our findings demonstrate that this correlation, required for sustained fast-spiking, is in fact implemented in SIF, but not in MIF motoneurons at the protein level (Figs. 1, 2, 3, 10). Although these findings may account for the reported lower threshold and reduced firing level of MIF motoneurons (Hernández et al. 2019), it cannot exclude a potential generation of bursts through the presence of other Kv subunits alongside Nav1.6 channels, as reported for Purkinje cells (Khavandgar et al. 2005; McKay and Turner 2004; Zagha et al. 2008). The hypothesis that the burst activity of MIF motoneurons is achieved through a set of different ion channel subunits complies with possibly distinct evolutionary origins of MIF and SIF motoneurons (Horn and Straka 2021; Walls 1962).

In addition, Kodama et al. (2020) reported a covarying expression of neurofilament proteins along with ion channels that are characteristic for the fast-spiking gene module. These features also correlate with axon caliber differences and even the regulation of dynamics and sustainability of transmitter release (Kodama et al. 2020). These findings also agree with the observation that MIF motoneurons lack non-phosphorylated neurofilaments (NP-NF) (Eberhorn et al. 2005). SIF motoneurons on the other hand co-express NP-NF (SMI-32), Kv1.1 and Kv3.1, and have larger axonal diameters indicative for sustained fast-spiking properties. Given that expression levels of neurofilaments covary with the axon caliber (Friede and Samorajski 1970; Lee and Cleveland 1996), a co-regulation of ion channel and neurofilament genes could account for a relationship between axon diameter as a proxy for high dynamics and fast-spiking capacity, as demonstrated for several other cell types (Perge et al. 2012).

#### Low voltage activated (T-type) calcium channels in MIF motoneurons and implications for muscle contractions

As members of a family of inward-permeating cation channels, low-voltage-activated (LVA) T-type calcium channels generally increase the excitability of cells (Weiss and Zamponi 2013). At resting membrane potential, these channels are typically inactive and therefore contribute only little to spike generation (Molineux, 2006; Weiss and Zamponi 2013). Upon release from a hyperpolarization, these channels depolarize the membrane to generate an LVA calcium spike and a rebound depolarization that drives a spike burst (post-inhibitory rebound burst) (Molineux et al. 2006). Therefore, T-type calcium channels are associated with regulating the excitability and pace-making at subthreshold voltage levels (Weiss and Zamponi 2013).

Selective expression of Cav3.1 channel, as observed in MIF motoneurons (Fig. 10), was shown to be sufficient for a rebound discharge (Molineux et al. 2006). On the other hand, cerebellar stellate and basket cells which express only Cav3.2 or Cav3.3 do not generate a rebound discharge under normal conditions, but only upon blockade of potassium channels (Molineux et al. 2006). The Cav3.3 subunit also contributes to a rebound discharge when co-expressed with potassium channels (Molineux et al. 2006). Taken together, post-inhibitory rebound depolarization generated by the Cav3.1 channel could be responsible for the initial membrane depolarization and bursting in MIF motoneurons, with a different mechanism than that implemented in SIF motoneurons. Accordingly, the excitability and bursting behavior of SIF motoneurons are likely regulated by a combination of Kv channels and more efficient glutamatergic transmission. These differences in intrinsic membrane properties could explain why MIF motoneurons have lower recruitment thresholds and firing rates, while at the same time they are apparently able to generate spike bursts (Hernández et al. 2019). The lack of Kv3.1 and Kv1.1 channels, combined with Cav3.1 channel expression, suggests that the population of MIF motoneurons in this study corresponds to the early recruited motoneuron population with low sensitivity and extended tonic firing. In contrast SIF motoneurons exhibit brief bursts followed by a less persisting tonic firing (Davis-López de Carrizosa et al. 2011; Gonzalez-Forero et al. 2003).

T-type calcium channels, by causing action potential-independent sustained Ca^2+^ elevations near the resting membrane potential, control a multitude of cellular and physiological functions, such as (low-threshold) vesicular exocytosis (Weiss and Zamponi 2013). As a result, MIF motoneurons might release significant amounts of neurotransmitters from their axonal boutons around the resting membrane potential (Weiss and Zamponi 2013), especially with respect to the window current of T-type calcium channels (Perez-Reyes 2003). When activation and inactivation curves of T-type calcium channels overlap, a sustained influx of Ca^2+^ through the channels around the resting membrane potential is possible for up to minutes, where a significant proportion of channels is open, but not yet completely inactivated (Perez-Reyes 2003). This phenomenon, i.e., “window current”, could facilitate low-threshold acetylcholine release onto multiply-innervated muscle fibers for an extended time. This is in contrast to singly-innervated fibers, where the discharge of SIF motoneurons is only brief, but at a considerably higher spike rate (Hernández et al. 2019), and therefore provokes a larger quantal transmitter release per time. Based on this interpretation, selective Cav3.1 channel expression in MIF motoneurons could account for a role in the activation of all eye movement types, albeit with lower sensitivity and with a predominant contribution to the maintenance of an elevated muscle tone (Hernández et al. 2019; Wasicky et al. 2004).

### Presynaptic inputs and intrinsic membrane properties of internuclear neurons in comparison to SIF motoneurons

Internuclear neurons of the abducens nucleus, which use glutamate and/or aspartate as transmitter (Nguyen and Spencer 1999), receive patterns of synaptic inputs from premotor sources similar to adjacent abducens motoneurons (Spencer and Sterling 1977; Straka and Dieringer 1993). While motoneurons are cholinergic and project to the lateral rectus muscle, INTs project to the medial rectus subgroup of the contralateral oculomotor nucleus, enabling horizontal conjugate eye movements (Baker and Highstein 1975; Büttner-Ennever and Akert 1981; Ugolini et al. 2006). Following electrical stimulation of the vestibular nerve in the cat and frog, both abducens motoneurons and internuclear neurons display disynaptic EPSPs on the contralateral and IPSPs on the ipsilateral side (Baker and Highstein 1975; Straka and Dieringer 2004). Monosynaptic excitatory premotor commands for saccades arise from the ipsilateral pontine reticular formation (Highstein et al. 1976; Strassman et al. 1986). Interestingly, the sources of synaptic inputs received by INTs are similar to those of SIF motoneurons, but not to those of MIF motoneurons (Ugolini et al. 2006). This suggests that signals mediated onto MIF abducens motoneurons obviously find no correspondence in an internuclear pathway that interconnects the respective synergistic subtypes of lateral and medial rectus motoneurons.

Internuclear neurons facilitate the conjugacy of both eyes by mediating a signal copy to the oculomotor nucleus because this neuronal population exhibits the same burst-tonic firing pattern during horizontal eye movements (Fuchs et al. 1988). Moreover, INTs fire slightly before and with a somewhat higher intra-burst frequency than abducens motoneurons (Delgado-García et al. 1977), which facilitates precise phase relationships of lateral rectus and medial rectus muscle contractions (Davis-López de Carrizosa et al. 2011; Pastor and González-Forero 2003). The enhanced excitability and earlier recruitment of INTs potentially derives from morpho-physiological adaptations. Notably, the distinct behavior of INTs might be related to a higher density of synaptic inputs, as compared to SIF motoneurons (Highstein et al. 1982). Indeed, the present study has demonstrated a higher density of presumed inhibitory synaptic contacts onto the somata of INTs, compared to those on SIF motoneurons (Figs. 6b, c, 7b, c). However, only the density of GAD-immunopositive puncta was significantly higher, while the synaptic density of other transmitter types on SIF motoneurons and INTs were similar, in agreement with electron microscopic observations (Spencer and Sterling 1977). On the other hand, consistently stronger Kv3.1b- and PN-immunolabeling were found in INTs, as compared to SIF motoneurons (Figs. 1f, 2c), despite a higher density of glutamatergic synapses. This could explain an enhanced excitability and intra-burst frequency of INTs (Delgado-García et al. 1977).

A major difference between INTs and motoneurons emerging from the current study was the strong immunolabeling of NMDAR1 in the former cell type (Fig. 4f). Intense somatic and dendritic labeling of INTs with the NMDAR1 antibody assigns a distinct NMDAR-mediated excitation to these neurons in comparison to motoneurons. NMDA receptors mediate slower and more prolonged responses, whereas AMPA receptors provoke more rapid and transient responses (Traynelis et al. 2010). The intense NMDAR1 expression by INTs in comparison to motoneurons could assist in spike burst generation, as NMDA receptors were shown in cat abducens motoneurons to evoke a burst at threshold level followed by a stable repetitive firing (Durand et al. 1987).

### Differential control of eye movements by extraocular motoneuron and muscle fiber types

Earlier work on extraocular muscles suggested the possibility that slow eye movements and gaze holding is achieved by slow-tonic (non-twitch) non-fatigable fibers, while fast eye movements such as saccades are produced by fast-twitch fatigable fibers, as observed in vertebrate skeletal muscles (Bormioli et al. 1980; Close and Hoh 1968; Henneman et al. 1965; Hoh 2020; Ugolini et al. 2006). However, the expression of numerous myosin heavy chain isoforms that define contraction characteristics in extraocular muscles suggests that the wide range of eye movements might be differentially controlled through the recruitment of several fiber types, rather than the often-emphasized bimodal distribution (Hoh 2020; Horn and Straka 2021). Indeed, apart from a classification according to the arrangement into an orbital and a global layer, extraocular muscle fibers can be subdivided into up to six types according to structural and histochemical properties such as fiber diameter, sarcoplasmic reticulum development, number of mitochondria and oxidative enzyme content (Shall and Goldberg 1992; Spencer and Porter 2006). In these analyses, global and orbital SIFs, as well as orbital MIFs, are arranged along a spectrum of fast/slow and fatigable/fatigue-resistant properties, whereas global MIFs are positioned at the non-twitch/slow-tonic end containing the slowest myosin heavy chain isoforms (Close and Hoh 1968; Hernández et al. 2019; Hoh 2020; Matyushkin 1964).

## Conclusion

Distinct labeling patterns of ion channel and neurotransmission-related proteins were encountered for MIF and SIF motoneurons in the abducens and trochlear nuclei of macaque monkeys, in agreement with putative differences in physiological properties. These findings complement previous reports with regard to several functional aspects: (i) MIF motoneurons, histologically identified by ChAT-immunopositivity and lack of PNs likely correspond to global MIF motoneurons identified in cats by lower spike thresholds and firing rates. (ii) Although motoneurons along the entire spectrum of firing rates and activation thresholds are active during all eye movement types, the clear histological distinction including innervation pattern between SIF and MIF motoneurons suggests a differential distribution of electrophysiological properties. (iii) The wide range of motoneuronal activity might be achieved through a combination of varying density in transmitter phenotype, receptor expression pattern and ion channel composition. The immunohistochemical dissection performed in the current study thus represents a major step in linking morphological and biochemical features with known physiological and pharmacological properties, providing the basis for an assessment of corresponding aspects of homologous nuclei involved in human eye movement pathologies.

## Data Availability

The datasets generated during this study are available from the corresponding author on reasonable request*.*
